# PuF, an antimetastatic and developmental signaling protein, interacts with the Alzheimer’s amyloid-β precursor protein via a tissue-specific proximal regulatory element (PRE)

**DOI:** 10.1186/1471-2164-14-68

**Published:** 2013-01-31

**Authors:** Debomoy K Lahiri, Bryan Maloney, Jack T Rogers, Yuan-Wen Ge

**Affiliations:** 1 Laboratory of Molecular Neurogenetics, Department of Psychiatry, Institute of Psychiatric Research, Indiana University School of Medicine, 791 Union Drive, Indianapolis, IN, 46202, USA; 2 Department of Medical and Molecular Genetics, Indiana University School of Medicine, Indianapolis, IN, 46202, USA; 3 Neurochemistry lab, Department of Psychiatry, Massachusetts General Hospital, Harvard Medical School, Charleston, MA, 02129, USA; 4 Department of Psychiatry, Institute of Psychiatric Research, Indiana University School of Medicine, Indianapolis, IN, 46202, USA

**Keywords:** Amyloid precursor protein, Alzheimer’s disease, Cancer, Gene regulation, Gene transcription, Iron, Latency, nm23 nucleoside diphosphate kinase, Oncogenesis, PuF, SP1, Specificity protein 1, Transcription factor

## Abstract

**Background:**

Alzheimer’s disease (AD) is intimately tied to amyloid-β (Aβ) peptide. Extraneuronal brain plaques consisting primarily of Aβ aggregates are a hallmark of AD. Intraneuronal Aβ subunits are strongly implicated in disease progression. Protein sequence mutations of the Aβ precursor protein (APP) account for a small proportion of AD cases, suggesting that regulation of the associated gene (*APP*) may play a more important role in AD etiology. The *APP* promoter possesses a novel 30 nucleotide sequence, or “proximal regulatory element” (PRE), at −76/−47, from the +1 transcription start site that confers cell type specificity. This PRE contains sequences that make it vulnerable to epigenetic modification and may present a viable target for drug studies. We examined PRE-nuclear protein interaction by gel electrophoretic mobility shift assay (EMSA) and PRE mutant EMSA. This was followed by functional studies of PRE mutant/reporter gene fusion clones.

**Results:**

EMSA probed with the PRE showed DNA-protein interaction in multiple nuclear extracts and in human brain tissue nuclear extract in a tissue-type specific manner. We identified transcription factors that are likely to bind the PRE, using competition gel shift and gel supershift: Activator protein 2 (AP2), nm23 nucleoside diphosphate kinase/metastatic inhibitory protein (PuF), and specificity protein 1 (SP1). These sites crossed a known single nucleotide polymorphism (SNP). EMSA with PRE mutants and promoter/reporter clone transfection analysis further implicated PuF in cells and extracts. Functional assays of mutant/reporter clone transfections were evaluated by ELISA of reporter protein levels. EMSA and ELISA results correlated by meta-analysis.

**Conclusions:**

We propose that PuF may regulate the *APP* gene promoter and that AD risk may be increased by interference with PuF regulation at the PRE. PuF is targeted by calcium/calmodulin-dependent protein kinase II inhibitor 1, which also interacts with the integrins. These proteins are connected to vital cellular and neurological functions. In addition, the transcription factor PuF is a known inhibitor of metastasis and regulates cell growth during development. Given that APP is a known cell adhesion protein and ferroxidase, this suggests biochemical links among cell signaling, the cell cycle, iron metabolism in cancer, and AD in the context of overall aging.

## Background

A diagnostic feature of Alzheimer’s disease (AD) is aggregation of toxic amyloid β peptide (Aβ) into extracellular plaques, suspected of causing or contributing to disease progression [1,2]. In addition, intracellular Aβ has been implicated as having a pathological role in AD and Down syndrome [3], and Aβ may function as a transcription factor (TF) [4,5]. Aβ is cleaved from a larger precursor protein (APP) by a process involving two secretase enzymes, β-secretase and γ-secretase [2]. Hyperphosphorylated τ protein and α-synuclein also have a likely role in AD etiology [6-8], and apolipoprotein E (*APOE*) is linked to a large proportion of cases of AD both by genetic [9,10] and cholesterol-related functional studies [11]. We hypothesize that unusually high production of Aβ significantly contributes to AD, and this aberrant Aβ production can result from unusually high APP gene (*APP*) expression, particularly in a tissue and cell-type specific manner.

Several groups have studied the 5^′^-flanking region of the *APP* gene, including its promoter [12-16]. Serial deletion analysis has shown that the *APP* promoter [14,17] and 5^′^-UTR [18] contain several regulatory elements that are likely to modulate transcriptional activity. In addition to proximal regulatory regions, two upstream sequences have been identified that regulate the gene’s expression [14], including one that has been shown to generally stimulate APP production [19]. The *APP* promoter is regulated by a variety of factors. It can be stimulated by nerve growth factor, fibroblast growth factor, and interleukin-l [20,21], and copper depletion downregulates its activity [22]. A link between *APP* gene regulation and pathologies such as AD has been shown, for example, by characterization of two *APP* promoter polymorphisms associated with the pathogenesis of some forms of AD [23]. The more upstream of these two polymorphic sites may function as a target site for Aβ acting as a transcription factor [4,5].

Our group has examined regulatory regions of important AD-associated genes, including *APOE*[24,25] and microtubule-associated protein τ (*MAPT*) [26]. We have also previously characterized a deletion series of the *APP* promoter in eight different cell lines from five different tissue types in a chloramphenicol acetyltransferase (CAT) reporter construct [27]. We discovered that a novel 30 nucleotide (nt) sequence of −76 to −47 from the +1 transcription start site (TSS) had differential effect depending upon cell line. In human kidney epithelial cells, deletion of this element resulted in 50-fold reduction of CAT reporter gene activity. In human SK-N-SH neuroblastoma (NB) cells, deletion resulted in a 3 to 4-fold gain of reporter gene activity, the greatest NB cell expression for all clones of the deletion series. We thus termed this region the “proximal regulatory element” (PRE) of the *APP* gene. We examined the nature of DNA-protein interaction with this DNA fragment by gel electrophoretic mobility shift assay (EMSA or gel shift). Notably, use of the PRE as a probe in EMSA showed evidence of DNA-protein interaction with this sequence in multiple cell line nuclear extracts and in mouse brain tissue nuclear extract [27]. However, the specific nature of nuclear proteins that interact with the PRE in different cell types was not determined at that time.

We continue our work on the PRE by exploring specific DNA-protein interactions in EMSA, competition EMSA, and antibody-supershift EMSA. Functional effects were measured by creation of a library of mutant PRE sequences within a previously constructed [17]*APP*-CAT fusion clone. While we had previously shown that the PRE interacts with nuclear proteins in a tissue-type specific manner, we herein identified TFs that were likely bind to the PRE, specifically activator protein 2 (AP2), nm23 nucleoside diphosphate kinase/metastatic inhibitory protein (PuF), and specificity protein 1 (SP1). We also characterized quantitative and qualitative effects of mutating the PRE vs. DNA-protein interaction in EMSA vs. both NB and rat neuronal pheochromocytoma (PC12) cells and cell nuclear extracts. Functional assays of PRE mutation effects were performed by transiently transfecting the mutant-CAT clones into human neuroblastoma and rat neuronal pheochromocytoma cell cultures. We discovered specific, significant mutation-dependent function differences. We compared the EMSA results to effects of the same mutations in functional mutant-CAT clone transfection assays. Altering the PRE’s ability to bind TF corresponded to functional changes in promoter activity in a cell line-specific manner.

We determined that PuF and SP1 are candidates for regulation of the *APP* gene through the PRE. PuF’s better-known function is as an inhibitor of metastasis [28]. SP1 activity in *APP* regulation has already been well demonstrated [29-33]. SP1 sites have been located in both the promoter [13,32,34] and 5^′^-UTR portions of the *APP* 5^′^-flanking region [35,36]. Our data led us to propose that SP1 and PuF act antagonistically through the PRE, with SP1 stimulating and PuF repressing *APP* transcription. These two TF sites can be subject to natural variation in the human genome, as they cross a known single nucleotide polymorphism (SNP) [37]. The PRE sequence contains sites for DNA methylation and oxidation, suggesting the site may be vulnerable to environmentally-mediated epigenetic modification. Should such interference with PuF regulation of *APP* increase risk of AD, it would be another similarity between etiology of sporadic/idiopathic neuropsychiatric disorders and oncogenesis, extending our previous work in developing the latent early-life associated regulation (LEARn) model [38,39] of idiopathic neuropsychiatric disorders.

## Results

### Putative transcription factor sites within the PRE

The PRE with an additional 10nt flanking sequence at either end was used with TESS [40] and MatInspector [41] to probe the TransFac database [42]. Restricting predicted sites to mammal TF matrices and eliminating redundant sites resulted in predicted affinities with several transcription factors (Table 1), including AP2, GATA binding proteins 1 (GATA1) and 2 (GATA2), two GC boxes, paired box gene 4-a (Pax4a), PuF, Epstein-Barr virus transcription factor R (R), SP1, and transcription elongation regulator 1 (mammal homologue to zeste, TCERG1). Truncated upstream stimulatory factor (USF)1 and USF2 sites were also found at the 3^′^-end of the PRE. The GC box is associated with the binding of several transcription factors, including SP1, basic transcription element binding protein (BTEB)1, BTEB2, and msh homeobox 1 (Msx1). 

**Table 1:** Predicted TF sites in the PRE

**Factor**	**Sequence within PRE**^**a**^	**MW (kDa)**^**c**^
AP2	GGGGTGGGCCG	32, 40, 47, 48, 49, 50, 51, 52
BTEB1^e^	GAGCGG	
	GGCCGG	
BTEB2^e^	GAGCGG	25
	GGCCGG	
GATA1	GGGTGGGC	43, 51
	CCGGATCAGc	
GATA2	CCGGATCAGc	50
Msx1^e^	GAGCGG	31
	GGCCGG	
Pax4α	GGGGTGGGCCGG	38
PuF	GGGTGGG	17
R	GTGCCGAGCGGGGTGGGC	67
SP1^b,e^	GGGTGG	81, 95, 105
	TGGGCCGGATCAGctg	
	GAGCGG	
	GGCCGG	
TCERG1	CGAGTG	122, 124^d^
	CGAGCG	
USF1	GGATCAGctgactc	34, 43, 55
USF2	GGATCAGctgactc	37, 44

^a^All sequences presented in the same orientation as the PRE. Partial sites are also listed, with bases that extend past the PRE indicated by dashes.

^b^Two distinct SP1 sites were identified in the wildtype PRE sequence in addition to the GC Box.

^c^Unless otherwise indicated, MW are for isoforms listed in the TransFac database [42].

^d^MW is calculated from predicted genomic amino acid sequence.

^e^One of several potential GC Box-binding factors.

### DNA-protein interaction of the PRE by EMSA varies among tissue types and cell line conditions

**Figure 1:**
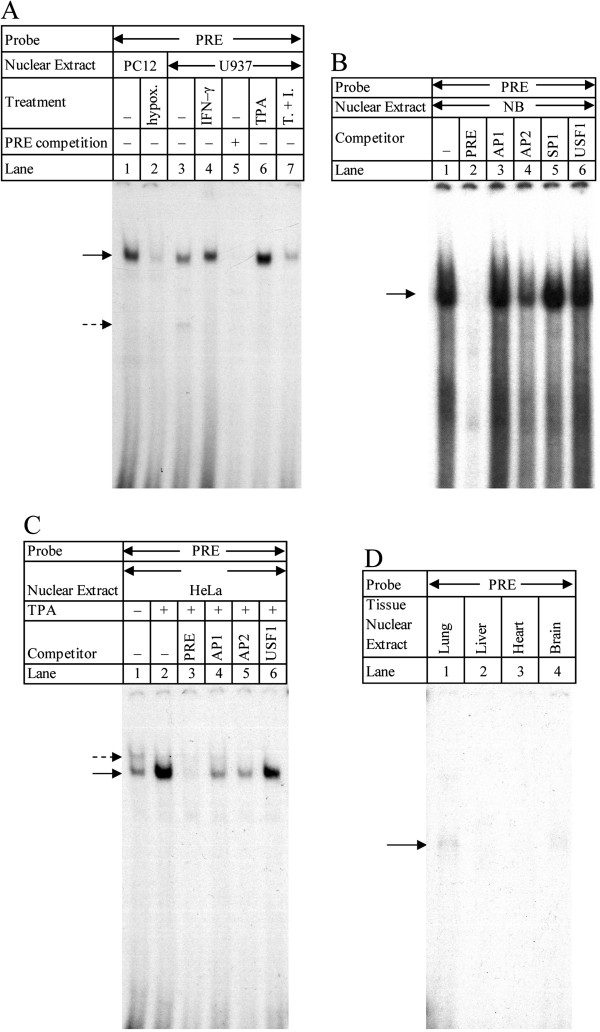
**EMSA in stimulated and unstimulated cell culture nuclear extracts and vs. various transcription factor binding oligomers. A**. Radiolabeled PRE was incubated with PC12 or U937 nuclear extracts. PC12 extracts were either normal (lane 1) or from cells subject to hypoxia (lane 2). U937 extracts were either normal (lanes 3, 6) or from INF-γ induced (lane 4), TPA-induced (lane 6), or TPA + INF-γ induced cells (lane 7). In addition, one sample of normal U937 extract was incubated both with labeled and 200x molar excess of unlabeled PRE (lane 5). Arrows indicate major DNA-protein interactions. **B**, **C***.* The PRE fragment from plasmid pβXbB was radiolabeled and incubated in NB or HeLa cell nuclear extracts with or without 200x molar excess competition against oligomers known to bind specific transcription factors. DNA-protein interactions are indicated with arrows. B. NB cell nuclear extracts. No competition (lane 1), competition vs. unlabeled PRE (lane 2), competition vs. unlabeled oligomers that bind AP1 (lane 3), AP2 (lane 4), SP1 (lane 5), or USF1 (lane 6). **C**. HeLa (lane 1) or TPA-stimulated HeLa (lanes 2–6) nuclear extracts. Reactions were subjected to no competition (lanes 1–2) competition vs. 200x molar excess of unlabeled PRE fragment (lane 3), AP1-binding oligomer (lane 4), AP2-binding oligomer, or USF-binding oligomer (lane 5). **D**. EMSA of the PRE in four human tissue nuclear extracts. The DNA fragment corresponding to the PRE (−76/-47) was purified from plasmid pβXbB, radiolabeled, and incubated in nuclear extracts from human lung (lane 1), liver (lane 2), heart (lane 3), or brain (lane 4). DNA-protein interaction band is indicated by arrow. Unbound probe ran at the bottom of the gel (not shown).

To investigate cell type specificity of PRE-protein interactions, we used the PRE fragment in EMSA with nuclear extracts from PC12 and human cervical epithelial (HeLa), SK-N-BE neuroblastoma (NB), and histiocytic lymphoma (U937) cells (Figure 1A–C) and with nuclear extracts from human tissues (Figure 1D). PC12 extracts (Figure 1A) were obtained from both normal and hypoxic cells (lanes 1–2). U937 extracts (Figure 1A) included nuclei from untreated cells and from cells treated with interferon (IFN)-γ, 12-O-tetradecanoylphorbol-13-acetate (TPA), or TPA + IFN-γ (lanes 3–7). In addition, to test specificity of PRE binding with untreated U937 extracts, we also competed the PRE/U937 EMSA against 200x molar excess unlabeled PRE fragment (lane 5). Nuclear extract EMSA showed definite interaction between the PRE and both PC12 and U937 extracts. This interaction was competed away with excess PRE in U937 extracts. DNA-protein interactions could be altered by specific treatment of cells from which extracts were taken. Hypoxia greatly reduced the observed interaction in PC12 extracts (lane 2). Treatment with the cytokine IFN-γ may have slightly increased interaction in U937 extracts (lane 4), but TPA treatment seems to have produced a stronger result (lane 6). Combined TPA+IFN-γ treatment (lane 7) reduced DNA-protein interaction levels, suggesting that more than one pathway may be involved in interaction with the PRE and that these interactions may compete with each other.

To narrow down candidate transcription factors predicted by TESS we conducted EMSA with 200x molar excess of unlabeled oligomer pairs known to bind the transcription factors activator protein 1 (AP1), AP2, and SP1, respectively. In addition, to demonstrate that the truncated USF1 site at the 3^′^-end of the PRE is not active, we competed against an unlabeled commercial oligomer pair known to bind USF1 (Figure 1B, C). Two assays were carried out under the same conditions, one each with nuclear extracts from NB (Figure 1B) and HeLa (Figure 1C) cells, respectively. In NB extracts, competition against AP1, SP1, and USF1 binding oligomer pairs (lanes 3, 5, 6) showed no difference with the uncompeted lane sample (lane 1). Competition with unlabeled PRE eliminated DNA-protein interactions (lane 2), while competition with AP2-binding oligomer pair reduced but did not eliminate interaction, suggesting that PRE interaction in neuronal cells may operate through more than one pathway or at least with more than one potential transcription factor.

Competition EMSA in HeLa extracts was in TPA-treated extracts (Figure 1C, lanes 2–6). Comparing untreated HeLa nuclear extracts with TPA-treated extracts revealed that TPA induction changed DNA-protein interactions with the PRE (Figure 1C, lanes 1–2). Competition with the PRE nearly eliminated DNA-protein interaction (lane 3). Competition with oligomer pairs that bind AP1 (lane 4) and AP2 (lane 5) partially reduced DNA-protein interaction. Competition with the USFl-binding oligomer pairs showed little difference to uncompeted TPA-treated extracts (lane 6). These results indicate that the PRE may interact with AP2, at the very least, but AP2 is neither the sole interaction partner for the PRE in neuronal cells nor under phorbol ester induction.

To directly explore tissue specificity of PRE interaction with factors present specifically in whole-tissue human organs in addition to cell cultures, we also carried out EMSA of the PRE fragment in human brain, heart, liver, and lung nuclear extracts. Two of the four extracts tested formed DNA-protein complexes with the PRE, specifically lung and brain extracts (Figure 1D lanes 1, 4). This suggests that the PRE is likely to have tissue-specific function *in vivo*, in addition to what may be suggested by cell culture results, and that the brain is at least one center of such affinity.

### DNA-protein interactions in NB nuclear extracts is TF specific by gel supershift EMSA

**Figure 2:**
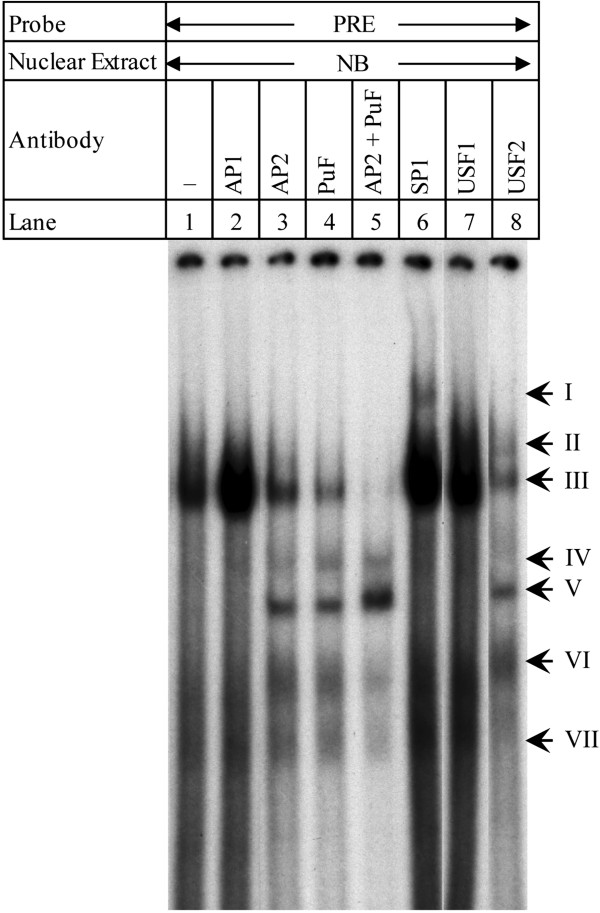
**Antibody supershift EMSA of PRE in NB nuclear extracts.** Radiolabeled PRE oligomer pairs were incubated with NB cell nuclear extracts (lane 1) and with extracts in the presence of antibodies against transcription factors AP1 (lane 2), AP2 (lane 3), PuF (lane 4), AP2 + PuF (lane 5), SP1 (lane 6), USF1 (lane 7), or USF2 (lane 8). Arrows indicate seven major bands.

For further information on specific nuclear factors that would interact with the PRE, we performed supershift EMSA with radiolabeled PRE oligomer pairs in NB nuclear extracts (Figure 2). Extracts were either untreated or co-incubated with antibodies against nuclear factors AP1, AP2, PuF, SP1, or combined AP2 + PuF. As negative controls, we incubated NB extracts with labeled PRE oligomer pairs and antibodies against USF1 or USF2.

Incubation of PRE oligomers with NB extracts was not altered by co-incubation with anti-AP1 (lanes 1–2), primarily consisting of a single band. As predicted, anti-AP2 and anti-PuF each altered binding of the PRE with NB extracts (lanes 3–4). In addition to reducing the major DNA-protein interaction of un-competed PRE (band III), new DNA-protein interactions (bands V and VI) became visible when anti-AP2 was co-incubated. Co-incubation with anti-PuF produced a similar pattern to anti-AP2, with an additional band (IV). Of particular interest is that co-incubation with both antibodies reduced DNA-protein interaction more than did incubation with either antibody alone, and completely eliminated the interaction at band III (lane 5).

Co-incubation with anti-SP1 produced a more classic “shift” response (lane 6, band I). Co-incubation with anti-USF1 (lane 7) did not result in any changes. Co-incubation with anti-USF2, on the other hand, produced a response similar to that found with anti-AP2 or anti-PuF. In addition, band II appears, but this band may also be already present but masked by the breadth of band III in untreated reactions. Close examination of lane I indicates that the interaction at band VI may also be present in untreated reactions. Bands IV and V, on the other hand, do not appear when the PRE is incubated with NB extracts in the absence of antibody or in the presence of AP1, SP1, or USF1 antibodies. These results indicate that AP2, PuF, SP1, and USF2 are all potential candidates for interaction with the PRE.

### Southwestern blotting with the PRE shows different DNA-protein interactions among different cell lines and conditions

**Figure 3:**
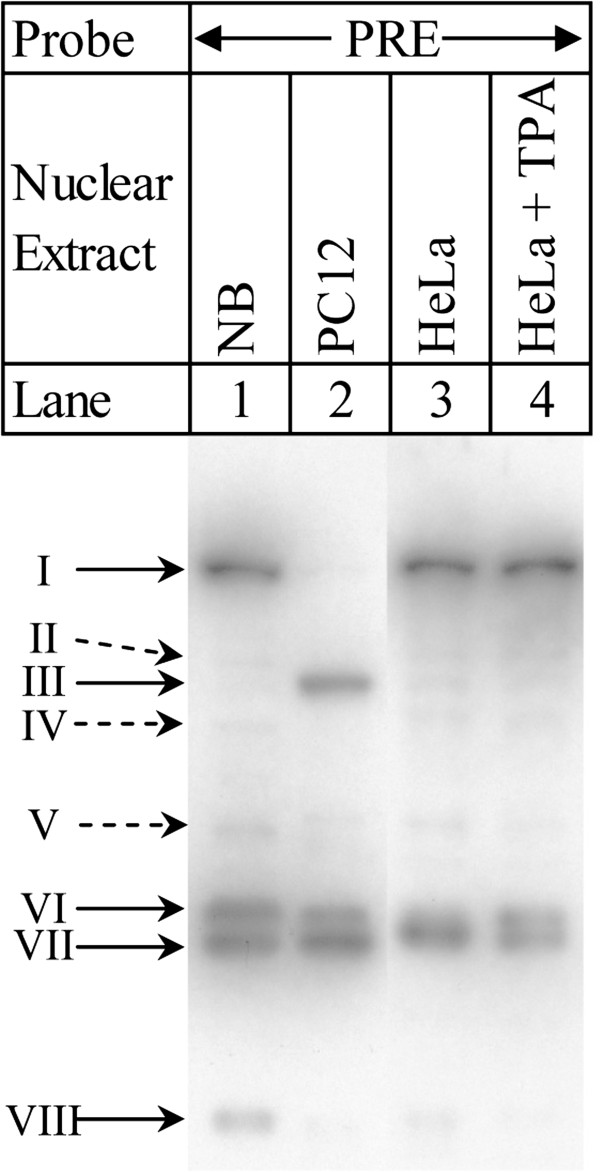
**Southwestern blot of PRE vs. cell line nuclear extracts.** Nuclear extracts from cell lines NB (lane 1), PC12 (lane 2), HeLa (lane 3), and HeLa treated with TPA (lane 4) were separated on 10% SDS-PAGE and transferred to nitrocellulose membrane. Membrane was probed with radiolabeled PRE oligomers. Blot photographs were size-equalized by use of the same lot of protein standards. Major bands are indicated with solid arrows. Minor bands are indicated with dotted-line arrows.

To ascertain sizes of proteins that participate in some of the observed interactions with the PRE, we performed southwestern blotting with nuclear extracts from NB, PC12, HeLa and TPA-treated HeLa cells (Figure 3). Probing with radiolabeled PRE oligomer pairs revealed that DNA-protein interactions varied among extracts and were subject to induction. NB nuclear extract (lane 1) had major interactions at approximately 92, 33, 32, and 23 kDa (the last an extrapolation outside the range of the size standard). In addition, faint bands appeared at approximately 64, 51, and 39 kDa. The PC12 nuclear extract (lane 2) also had a 33 kDa/32 kDa doublet but lacked a 92 kDa interaction. Instead, an interaction at approximately 60 kDa appears.

PC12 also had weaker interactions at 92, 39, and 23 kDa. HeLa nuclear extract (lane 3) shared interactions at approximately 92 and between 33 and 32 kDa, with fainter bands at 64, 60, 51, 39, and 23 kDa. Induction of HeLa with TPA (lane 4) resolved the 33/32 kDa band into a doublet and reduced the strength of weaker interactions. Comparison of the observed southwestern bands with molecular weights of predicted transcription factors suggests the possibility that several of the predicted candidate transcription factors may bind to the PRE (Table 2).

**Table 2:** Putative TF sites from southwestern blot based on estimated kDa of bands

**Band**^**a**^	**kDa**	**Nuclear extracts**^**b**^	**Corresponding TF**^**c**^
**I**	**92**	NB, HeLa, HeLa+TPA	SP1
II	64	NB, HeLa, HeLa+TPA	R
**III**	**60**	PC12, HeLa, HeLa+TPA	USF1, TCERG1
IV	51	NB, HeLa	USF1, AP2, GATA1, GATA2, TCERG1
V	39	NB	GATA1, USF1, AP2, Pax4a, USF2
**VI**	**33**	NB, PC12, HeLa, HeLa + TPA	USF1, AP2, Msx1
**VII**	**32**	NB, PC12, HeLa, HeLa + TPA	USF1, AP2, Msx1
**VIII**	**23**	NB	BTEB2

^a^Bands named in bold face are described as “major” in Figure 2.

^b^Nuclear extracts for which the band appears in the southwestern.

^c^TF correspondence is based on calculated band kDa ±10%.

These comparisons were based on a ±10% adjustment to the calculated southwestern band kDa. Of the potential DNA-TF interactions, several appear for all nuclear extracts. These are AP2, Msx1, and USF1. A few are unique to human neuroblastoma nuclear extracts, specifically Pax4a, USF2, and BTEB2. There is no signal that can be assigned to PuF subunits, which would be around 17kDa in weight. This may be an artifact of the specific gel’s polyacrylamide percentage. However, the band VI/VII doublet could correspond to PuF dimers. In general, the southwestern does not exclude conclusions of our EMSA-based assays.

### Mutating the PRE alters DNA-protein interaction

**Figure 4:**
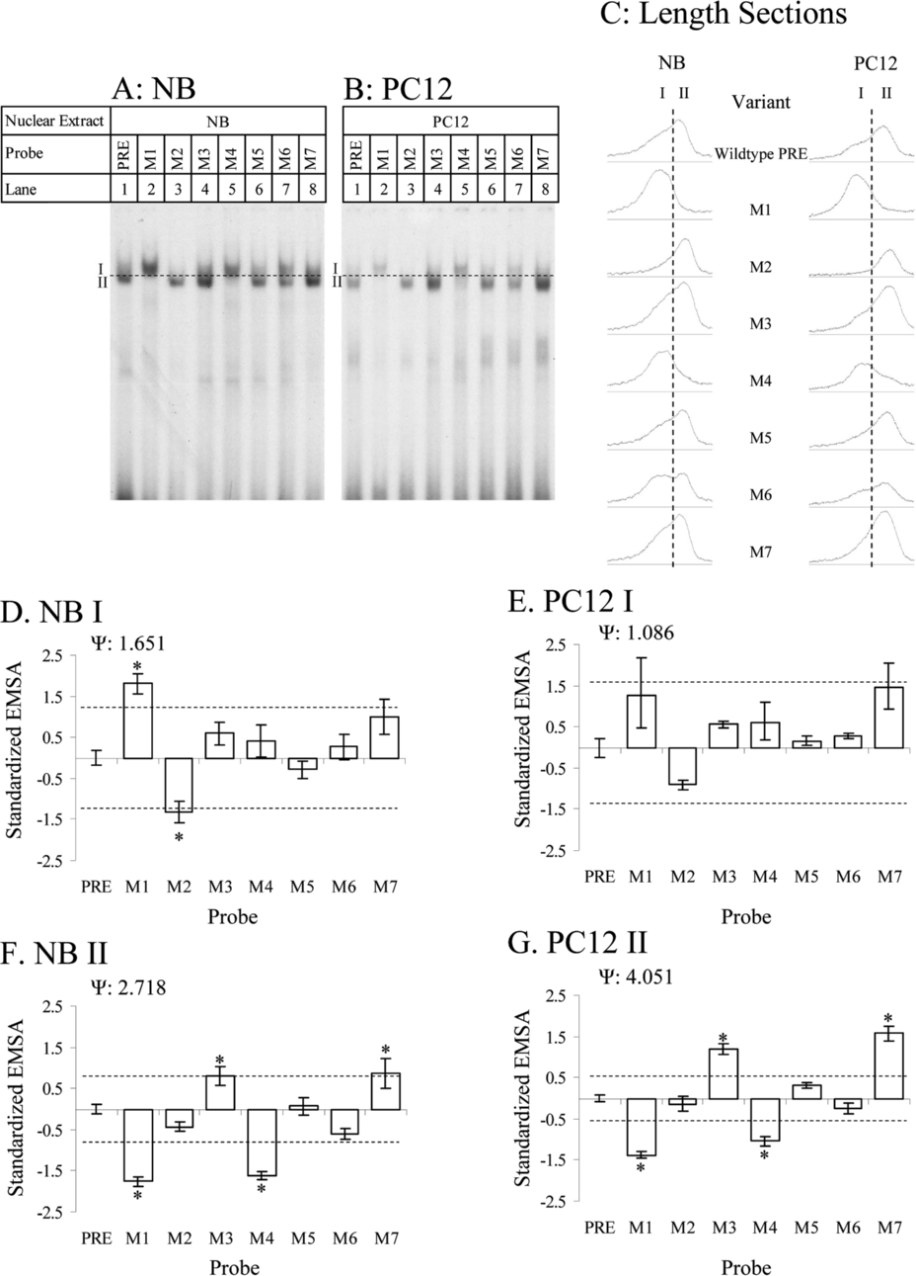
**EMSA of wildtype and mutant PRE oligomers and semiquantitative analysis of EMSA.** Wildtype and mutant PRE oligomers (M1-M7) were incubated with **A**. NB and **B**. PC12 nuclear extracts were analyzed on non-denaturing PAGE. Samples were analyzed in triplicate; figure presents a representative gel for each. Bands “I” and “II” as described in the main text are indicated. Dashed line indicates division made between band I and band II for densitometry purposes. **C**. Cross section of EMSA lanes. ImageJ software was used to evaluate densitometry of scanned films from EMSA of NB and PC12 nuclear extracts with various PRE variant oligomer pairs. Profiles of peaks for each PRE variant from a typical gel are shown. Dashed line indicates boundary between band I and band II for densitometry purposes. **D**–**G**. Densitometry readings for EMSA films were normalized to standard deviations and average readings for “wildtype” signal within each film as described in the main text. Normalized readings were compared to the wildtype reading by Dunnett’s multiple t. Means significantly different at p > 0.05 indicated by “*”. **D**. Band I, NB extract. **E**. Band I, PC12 extract. **F**. Band II, NB extract. **G**. Band II, PC12 extract.

To more specifically determine those sequences within the PRE that participate in gene regulation, we had synthesized a series of oligomer pairs that deleted one or more of the transcription factor sites we predicted to be in the PRE. These mutant oligomers were used for EMSA (Figure 4A, B) in both NB (lanes 1–8) and PC12 (lanes 9–16) cell nuclear extracts. Mutations resulted in both quantitative and qualitative alterations in DNA-protein interaction as determined by EMSA. Densitometry scans revealed that, at least for some mutants, such as M6, two partially overlapping bands appeared (Figure 4A, lane 7), and the wildtype oligomer pair has an extended trailing end to its peak, suggesting that wildtype binding is to more than one interaction partner. Semi-quantitative analysis, therefore, was done for all samples on each individual peak (division between peaks shown by dashed line in Figure 4A-C).

Starkly visible qualitative differences were restricted to mutants M1, M4 and M6. The M1 mutants’ predicted binding sites had lower affinity for AP2, GATA1, PuF, one of the two predicted SP1 transcription factor sites, and a downstream GC box. The M4 mutant is predicted to lose the shared GATA1/GATA2 site and the downstream SP1 site, The M6 mutant loses a predicted binding site for zeste homolog/TCERG1. In the case of the first two mutants, the major DNA-protein interaction product’s migration was reduced compared to the wildtype oligomer pair’s primary interaction product (Figure 4C). Analysis of relative migration rates for the wildtype oligomer pair showed that migration of the DNA-protein interaction products for M1 (lanes 2, 10) and M4 (lanes 5, 13) were significantly (p<0.05) reduced from the relative migration rate of wildtype oligomers (lanes 1, 9). The M6 mutation (lanes 7, 15) produced two major DNA-protein interactions in EMSA. The peak of the slower-running interaction was significantly (p<0.05) retarded in PC12 but not NB cells.

Samples were processed and analyzed in triplicate and films densitometrically scanned. Densitometry results were normalized by subtracting the mean peak value for a single film and dividing by standard deviation for the film. For data presentation purposes, this was adjusted by subtracting the standardized value of “wildtype”, which sets “wildtype” value to 0 (Figure 4D–G, Tables 3, 4). Adjusted data were analyzed by ANOVA followed by Dunnett’s two-tailed *t*. Hedges *g* standard pairwise effect sizes [43], using mean square error for pooled standard deviation, were calculated for each variant vs. wildtype PRE (Tables 3, 4). 

**Table 3:** Adjusted/normalized ELISA and EMSA signals in NB cells and extracts

**Variant**	**EMSA**^**a**^					
	**Band I**		**Band II**		**ELISA**^**a**^	
	**Rel. PRE = 0**	***g***	**Rel. PRE = 0**	***g***	**Rel. PRE = 1**	***g***
PRE	0.00 ± 0.18	0.00 ± 0.82	0.00 ± 0.11	0.00 ± 0.82	1.00 ± 0.17	0.00 ± 0.82
M1	1.81^c^ ± 0.24	3.24 ± 0.99/0.95	−1.76^c^ ± 0.11	−4.86 ± 1.29/1.31	0.37 ± 0.05	−3.12 ± 0.92/0.96
M2	−1.33^b^ ± 0.26	−2.39 ± 0.79/0.84	−0.43 ± 0.13	−1.18 ± 0.62/0.66	0.81 ± 0.12	−0.91 ± 0.60/0.63
M3	0.60 ± 0.27	1.07 ± 0.65/0.61	0.79^b^ ± 0.23	2.19 ± 0.80/0.76	1.69^b^ ± 0.13	3.40 ± 1.02/0.98
M4	0.40 ± 0.38	0.72 ± 0.62/0.59	−1.62^b^ ± 0.09	−4.48 ± 1.21/1.23	0.89 ± 0.02	−0.52 ± 0.58/0.60
M5	−0.28 ± 0.21	−0.51 ± 0.58/0.60	0.07 ± 0.22	0.19 ± 0.58/0.57	0.34^b^ ± 0.08	−3.24 ± 0.95/0.99
M7	0.27 ± 0.31	0.48 ± 0.60/0.58	−0.61 ± 0.12	−1.68 ± 0.68/0.73	0.16^b^ ± 0.12	−4.14 ± 1.13/1.16
M7	1.00 ± 0.42	1.78 ± 0.74/0.70	0.87^b^ ± 0.35	2.41 ± 0.84/0.79	1.04 ± 0.09	0.18 ± 0.58/0.57
	**F (p)**	**Ψ**^**d**^	**F (p)**	**Ψ**	**F (p)**	**Ψ**
ANOVA	9.79 (< 0.01)	1.65 ± 0.82/0.62	26.54 (< 0.01)	2.72 ± 1.09/0.97	21.20 (< 0.01)	2.43 ± 1.01/0.88

^a^Data is mean ± standard error of the mean (SEM). EMSA is normalized to wildtype = 0. ELISA is relative to wildtype = 1. SEM reported as (x/y as upper/lower) is asymmetrical due to noncentrality of standardized effect size measures [43].

^b^Signal significantly different from wildtype at p < 0.05.

^c^ELISA and EMSA signal both significantly different from wildtype at Bonferroni-adjusted p < 0.05.

^d^Ψ as estimate ± 95% confidence interval.

**Table 4:** Adjusted/normalized ELISA and EMSA signals in PC12 cells and extracts

**Variant**	**EMSA**^**a**^					
	**Band I**		**Band II**		**ELISA**^**a**^	
	**Rel. PRE = 0**	***g***	**Rel. PRE = 0**	***g***	**Rel. PRE = 1**	***g***
PRE	0.00 ± 0.23	0.00 ± 0.82	0.00 ± 0.1	0.00 ± 0.82	1.00 ± 0.11/0.09	0.00 ± 0.82
M1	1.25 ± 0.92/0.78	1.77 ± 0.74/0.69	−1.38^c^ ± 0.07	−5.56 ± 1.44/1.45	0.39^c^ ± 0.02	−6.84 ± 1.73
M2	−0.91 ± 0.11	−1.43 ± 0.65/0.69	−0.14 ± 0.18	−0.56 ± 0.58/0.60	0.67 ± 0.05/0.04	−2.05 ± 0.74/0.78
M3	0.56 ± 0.07	0.84 ± 0.63/0.60	1.20^b^ ± 0.14	4.82 ± 1.30/1.28	0.83 ± 0.18/0.12	−0.8 ± 0.59/0.62
M4	0.61 ± 0.48/0.44	0.91 ± 0.63/0.60	−1.04^c^ ± 0.11	−4.20 ± 1.15/1.17	0.45^c^ ± 0.01	−5.33 ± 1.39/1.4
M5	0.16 ± 0.12	0.25 ± 0.58/0.57	0.30 ± 0.07	1.23 ± 0.67/0.63	0.43^b^ ± 0.04/0.03	−5.69 ± 1.47/1.48
M6	0.27 ± 0.06	0.41 ± 0.59/0.58	−0.25 ± 0.13	−1.01 ± 0.61/0.64	0.43^b^ ± 0.01	−5.68 ± 1.47/1.48
M7	1.46 ± 0.58/0.52	2.04 ± 0.78/0.73	1.58^b^ ± 0.19	6.36 ± 1.62	1.03 ± 0.03	0.12 ± 0.58/0.57
	**F (p)**	**Ψ**^**d**^	**F (p)**	**Ψ**	**F (p)**	**Ψ**
ANOVA	4.23 (< 0.01)	1.09 ± 0.78/0.45	58.96 (< 0.01)	4.05 ± 1.49/1.42	29.96 (< 0.01)	2.89 ± 1.14/1.03

^a^Data is mean ± standard error of the mean (SEM). EMSA is normalized to wildtype = 0. ELISA is relative to wildtype = 1. SEM reported as (x/y as upper/lower) is asymmetrical due to noncentrality of standardized effect size measures [43].

^b^Signal significantly different from wildtype at p < 0.05.

^c^ELISA and EMSA signal both significantly different from wildtype at Bonferroni-adjusted p < 0.05.

^d^Ψ as estimate ± 95% confidence interval.

Analysis revealed differences between mutation effects on each band’s signal strengths. Specifically, for band I (the slower-migrating band), the M1 mutant had significantly higher adjusted signal than wildtype in NB (Figure 4D, Table 3) nuclear extract. M2 in NB and PC12 extracts had significantly lower adjusted signal than did wildtype (Figure 4E, Table 4). For the dominant wildtype PRE EMSA band (band II), M3 and M7 had significantly higher signal than wildtype in NB (Figure 4F, Table 3) and PC12 (Figure 4G, Table 4) nuclear extracts, while M1 and M4 had significantly reduced signal in NB and PC12 extracts.

### Mutating the PRE in a reporter gene system produces specific functional differences

**Figure 5:**
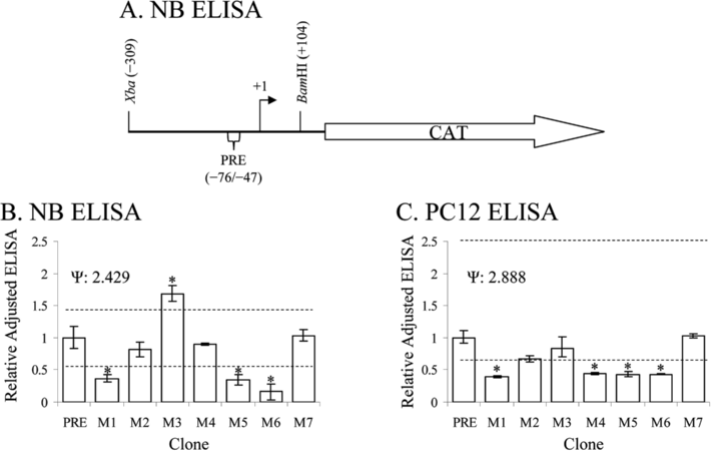
**ELISA of PRE variant CAT reporter clones in NB and PC12 cells.** Functional differences of PRE mutant clones (M1-M7) from wildtype were assayed in NB and PC12 cells. **A**. pβXbB were constructed and transfected into NB or PC12 cell cultures as described in the main text. Cultures were harvested and analyzed by CAT ELISA. ELISA signals were normalized to “wildtype” values and subject to Dunnett’s multiple t. Means significantly different at p > 0.05 indicated by “*”. **B**. ELISA of samples from NB cell cultures. **C**. ELISA of samples from PC12 cell cultures. Data presented is back-transformed from statistical analysis. Asymmetrical error bars and Dunnett’s t limits are due to Box-Cox transformation, when used.

To reveal connections between DNA-protein interactions and expression in mutations of the PRE, we constructed seven plasmids that mutated the PRE sequence identically to the mutant PRE oligomers. These cons-tructs were all based on pβXbB [17], which contains a promoterless *CAT* gene sequence under the control of an *Xba*I/*Bam*HI fragment of the rhesus monkey *APP* gene promoter and 5^′^-UTR, specifically −309/+104, with +1 being the transcription start site (Figure 5A). Clones were transfected into PC12 and NB cell cultures as described herein. 

ELISA results of the functional assay were adjusted for β-gal levels and made relative to wildtype = 1. Adjusted results were compared to wildtype by Dunnett’s *t*, and Hedges *g* was calculated for each pairwise comparison (Tables 3, 4). Functional mutation effects in NB cells (Figure 5B, Table 3) were distinct from PC12 (Figure 5C, Table 4). In NB cells three mutants (M1, M5, and M6) had reduced reporter expression compared to wildtype, and mutant M3, had greater adjusted reporter expression than wildtype. In PC12 cells, mutants M1, M4, M5, and M6 had significantly lower CAT protein levels than wildtype. Mutating predicted locations of putative transcription factor sites in the PRE significantly alters reporter gene expression. These results were consistent between cell lines for the M1 and M5 mutants.

### Semiquantitative EMSA and ELISA specifically correlate by PRE mutants

**Figure 6:**
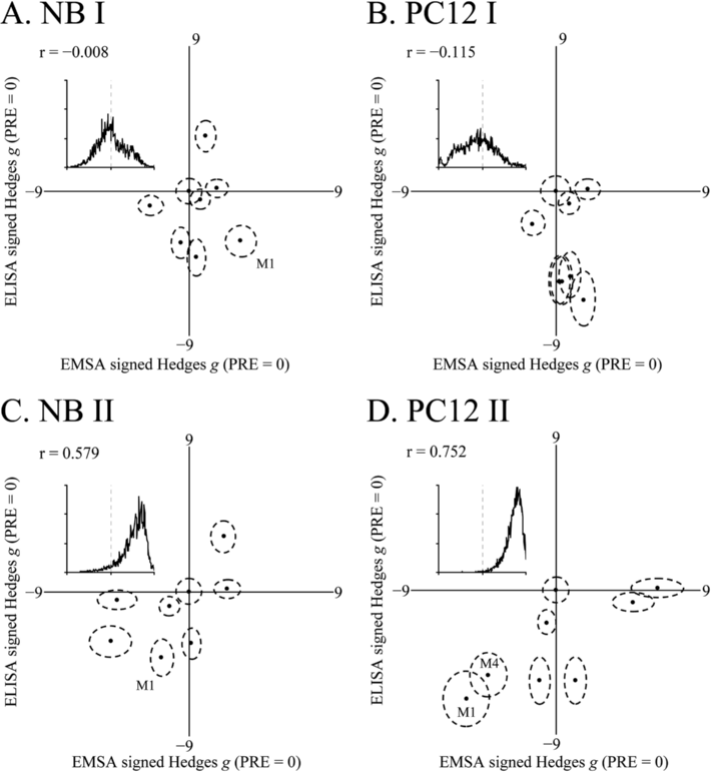
**EMSA vs. ELISA standardized effect sizes for PRE variants in NB and PC12 nuclear extracts and cultures.** EMSA and ELISA results were used to generate Hedges pairwise *g* of comparisons of each mutant to wildtype PRE. Data was plotted with EMSA *g* on X axis and ELISA *g* on Y axis. Samples where both EMSA and ELISA signals were significantly different from wildtype at Bonferroni adjusted p < 0.05 are indicated with corresponding mutant names. Correlations were bootstrapped (repeats = 100000), and frequency distributions of correlation coefficients are presented as insets. **A**. EMSA Band I vs. ELISA in NB extract/cells. **B**. EMSA Band I vs. ELISA in PC12 extract/cells. **C**. EMSA Band II vs. ELISA in NB extract/cells. **D**. EMSA Band II vs. ELISA in PC12 extract/cells.

We compared EMSA and ELISA results by meta-analysis of pairwise Hedges *g* for ELISA and EMSA results vs. wildtype (Figure 6). For both NB and PC12 cells/ nuclear extracts, much weaker correlations were found between EMSA band I and ELISA signal (Figure 6A, B). Stronger correlations existed between EMSA band II and ELISA signal in both cell lines (Table 5; Figure 6C, D). Significance was estimated as chance that r ≠ 0 by bootstrap (500,000 repetitions).

**Table 5:** Meta-analysis of PRE and mutant EMSA vs. ELISA

**Cell line**	**Correlation coefficient of ELISA vs.**		**% Bootstrapped correl. overlap**
	**EMSA band I (p)**	**EMSA band II (p)**	
NB	−0.008 (0.489)	0.579 (0.057)	39%
PC12	−0.115 (0.391)	0.752 (0.006)	16%

The percent overlap between the two bootstrap distributions within a single cell line was also calculated. The difference between correlations of EMSA I to ELISA vs. EMSA II to ELISA was more distinct in PC12 cells, with a 16% overlap between the two bootstrap distributions. In NB cells, the two bootstrap distributions overlapped by 34%. While there may be some indication of a direct relationship between EMSA band II signal and reporter ELISA results, we suggest caution in interpreting correlation meta-analysis. In addition to modest, at best, estimations of significance, EMSA bands I and II did not distinctly resolve on autoradiographs in many variants we studied, including wildtype (Figure 4). In addition, cell cultures used for ELISA and nuclear extracts used for EMSA did not come from the same specific cultures. Even with bootstrap estimation, they essentially represent correlations of averaged results from each assay. Nevertheless, we accept the possibility that some correspondence exists between altering the strength of the major DNA-protein interaction for the wildtype olig-omer pair (band II) and functional effects in reporter fusion assay.

### Mutating the PRE produces cell line-specific and inducible effects, as measured by EMSA

**Figure 7:**
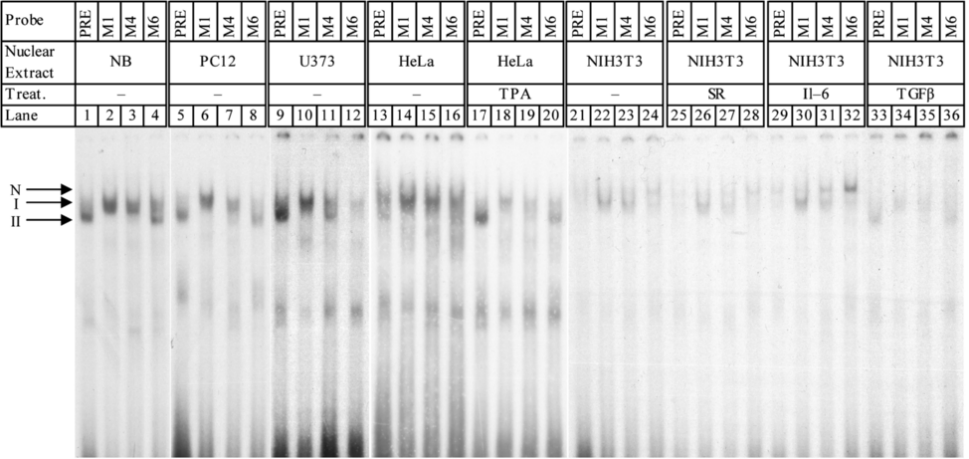
**EMSA of PRE and selected mutants in NB, PC12, HeLa, NIH3T3, and U373 nuclear extracts.** Wildtype oligomers (lanes 1, 5, 9, 13, 17, 21, 25, 29, 33) and oligomers for the M1 (lanes 2, 6, 10, 14, 18, 22, 26, 30, 34), M4 (lanes 3, 7, 11, 15, 19, 23, 27, 31, 35), and M6 (lanes 4, 8, 12, 16, 20, 24, 28, 32, 36) were labeled with γ−^32^P-ATP, incubated with NB (lanes 1–4) PC12 (lanes 5–8), U373 (lanes 9–12), HeLa (lanes 13–16), HeLa + TPA (lanes 17–20), NIH3T3 (lanes 21–24), NIH3T3 + serum restriction (lanes 25–28), NIH3T3 + IL6 (lanes 29–32), and NIH3T3 + TGFβ (lanes 33–36) nuclear extracts, and analyzed on nondenaturing PAGE, and gels subject to autoradiography.

To further investigate possible cell line/type-specific effects of the PRE, three mutants, specifically M1, M4, and M6, plus the wildtype PRE, were used as probes in EMSA with nuclear extracts from NB, PC12, human glioblastoma-astrocytoma (U373), HeLa or mouse embryo fibroblast (NIH3T3) cells. In addition, these oligomers were assayed with EMSA with nuclear extracts from HeLa, or NIH3T3 cells that had been subject to induction conditions, such as TPA treatment, serum starvation or induction with cytokines such as interleukin-6 (IL6) or transforming growth factor β (TGFβ) (Figure 7). Three apparent bands, marked N (“null”/“zero”), I, and II, appeared among the various nuclear extracts, although no extract/mutant combination had all three bands. In nuclear extracts from NB cells (Figure 7, lanes 1–4), DNA-protein interactions were obtained as reported in 3.1. Wildtype oligomers produced a single strong interaction (lane I, band II). The M1 and M4 oligomer pairs each had an interaction that migrated more slowly than did the wildtype (lanes 2–3), while M6 had a mixed interaction, with bands that migrated both at the same rate as produced by wildtype and as by M1 (lane 4) oligomer pairs. The PC12 extracts (lanes 5–8) produced a pattern similar to that found with NB extracts, excepting that the DNA-protein interaction with M4 (lane 7) has a stronger “leading” edge to its band while the interaction with M6 has a weaker “trailing” peak in the band doublet (lane 8).

Interactions observed with U373 extracts (lanes 9–12) bore some resemblance to those observed with NB and PC12 nuclear extracts specifically for wildtype (lane 9) and M1 (lane 10). However, the M4 and M6 DNA-protein interactions (lanes 11, 12) were noticeably different from both NB and PC12. DNA-protein interactions seen with untreated HeLa nuclear extracts (lanes 13–16) did not apparently vary among PRE variants, but treatment of HeLa with TPA (lanes 17–20) resulted in a pattern of interactions that was more similar to those with NB and PC12 extracts.

Untreated HeLa (lanes 13–16) DNA-protein interactions were unlike those found in the other cell lines surveyed. They had an additional band that migrated more slowly than seen for other cell line nuclear extracts. Interaction was very weak with the wildtype oligomer (lane 13), and it appeared as a doublet. The M1 mutant’s interaction was a single band (lane 14), while M4 and M6 interactions were doublets (lanes 14–16).

Serum starvation of NIH3T3 (lanes 25–28) may have slightly reduced interaction with the M4 mutant (lane 27) but appeared to have no other effect. Treatment of NIH3T3 with IL6 (lanes 29–32) increases DNA-protein interaction with the M6 oligomer (lane 32, band I). Of particular interest, treatment of NIH3T3 with TGFβ (lanes 33–36) brought about a dramatic shift in DNA-protein interaction bands for the wildtype PRE and all three mutants. While interaction was visibly weaker than for other NIH3T3 based extracts, it very closely resembled that seen for normal NB (lanes 1–4) and PC12 (lanes 2–8) extracts.

### The PRE is well-conserved within the human genome and throughout eutherian mammals

**Figure 8:**
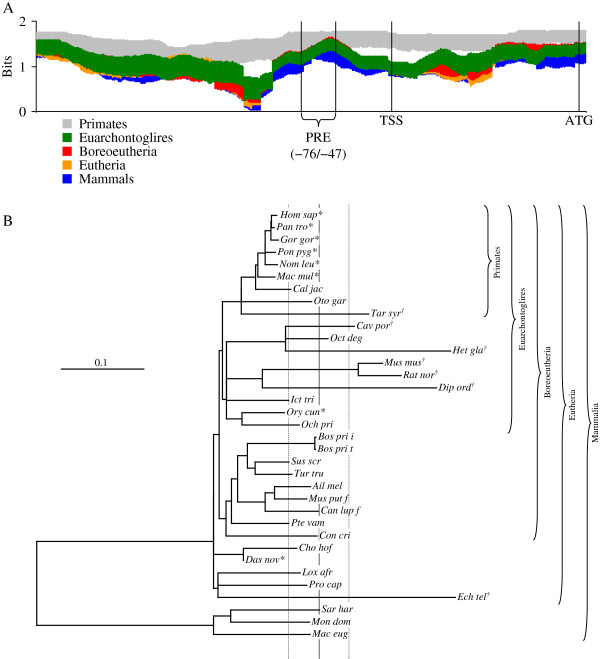
**Multiple sequence alignment of *APP *upstream regions and test of molecular clock.** DNA sequences from 20 mammal species were downloaded from GenBank and aligned with *WebPrank* as described in the main text. **A**. Average information content in a window of 100 nt and 95% confidence intervals were calculated for primates, euarchontoglires, boreoeutheria, eutheria, and mammals. **B**. Alignment was used as input for parsimony/FITCH combined phylogeny estimation as described in the main text. This produced a bifurcating non-ultrametric tree, although some branch lengths = 0. The tree was artificially rooted between marsupials and eutheria, and root-to-tip distances for each species calculated. Root-to-tip distances were compared to mean root-to-tip distance for the entire tree. Individual distances that differed from the mean distance (vertical solid line) ± the 95% (Bonferroni adjusted for 35 comparisons) confidence interval (vertical dashed lines) were counted as “significant”. Distances that significantly exceeded the mean indicated by “†”. Differences significantly lower than the mean indicated by “*”.

To explore the possibility that the PRE is well-conserved, we examined genomic sequences identified or putatively identified as proximally upstream of the *APP* gene of 35 mammal species that were aligned by *WebPrank*[44] as described herein. Information of the alignment was calculated in bits [45,46] and averaged in a window of 100 nt, which roughly corresponds to the distance between nucleosomes in chromosomal DNA as described herein. Examination of the alignment as a whole revealed that the PRE was well conserved compared to immediately flanking sequences. Adding non-primate sequences to the alignment did not change average information content immediately around the PRE (Figure 8A), but average information content for the alignment dropped off significantly (p <0.05, Bonferroni adjusted for 4 comparisons) when on either side of the PRE. Information inversely expresses homogeneity, since 100% homogeneity at a given base is maximum possible score (2 bits) and equal distribution of all four bases would be 0 bits. 

**Figure 9:**
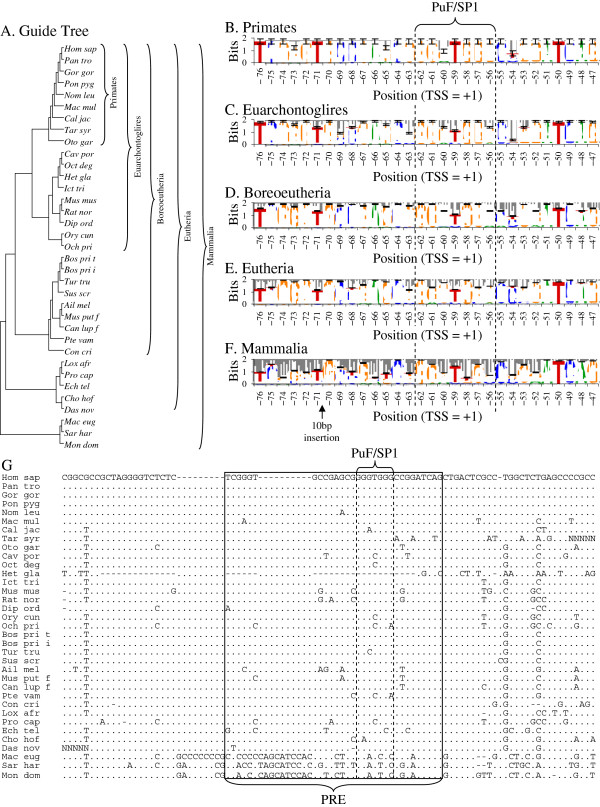
**Taxonomic guide tree, sequence logos for the PRE for five ascending clade levels from primates through mammals, and DNA alignment immediately surrounding the PRE. A.** The guide tree used for multiple sequence alignment with *WebPrank*[44]. Guide tree was assembled based on recent phylogenetic analysis of higher organization of mammals [46-49]. **B–F.** Sequence logos were calculated by conventional methods [50], with sequences taxonomically weighted as described in the main text. Membership in successively nested clades for each species is indicated. The location of the PuF/SP1 site is indicated for all logos. Error bars represent estimated standard deviations of R_i_. Logos were calculated for **B.** primates, **C.** Euarchontoglires, **D.** Boreoeutheria, **E.** Eutheria, and **F.** Mammals. Sequence specificity for the PRE and the PuF/SP1 site are preserved throughout placental mammals. **G.** The region of the alignment immediately around the PRE. The PRE is indicated with a solid box, and the PuF/SP1 site is indicated with dashed lines.

Phylogenetic comparison of the alignment (−326/+202 in the human sequence) led, as expected, to rejection of the molecular clock hypothesis (Figure 8B). DNA changes observed within the, admittedly short, region are unlikely to occur simply through neutral sequence drift. Of particular interest is that most of the primate sequences appeared to have a slower-than-typical rate of change vs. the overall tree while mouse and rat sequences had a faster-than-typical rate of change. Further detailed examination was done of the alignment region immediately surrounding the PRE (Figure 9). Alignment was guided by a tree (Figure 9A) based on current conventional mammal phylogeny [47-49], as suggested by the *WebPrank* protocol. Sequences were taxonomically weighted as described herein and homologies between the human sequence vs. other primates, euarchontoglires, boreoeutheria, eutheria, and all mammals were calculated for the aligned PRE, as were sequence logos [46] for these groups (Figure 9B–F). The PRE remains fairly stable throughout the eutheria, with no less than 92.3% weighted homology to the human sequence (Table 6). Adding marsupials to the estimation reduces homology to 73.8%. Greater aggregate distance from primates also associates with lower specificity within the PRE (Table 6)—total information content (essentially expressing sequence specificity in terms of bits) drops from 49.04 for primates (including humans) to 39.36 when all mammal sequences are considered (Table 6). Of particular interest, the overlapping SP1/PuF site within the PRE is also well conserved among placental mammals (Figure 9E). However, the site breaks down when marsupial sequences are included (Figure 9F). Marsupials also have a 9–10 base insertion within the PRE (Figure 9F–G). 

**Table 6:** **Analysis of *APP *proximal promoter region multiple sequence alignment**

	**n**	**PRE**		**PuF site**	
		**R**_**i**_^**c**^	**Homology**^**d**^	**R**_**i**_	**Homology**
Primates	9	49.04±3.81	95.1%±1.7%	11.31±0.93	98.2%±2.4%
Euarchontoglires	18	48.48±3.02	92.8%±1.8%	11.35±0.49	92.5%±4.2%
Boreoeutheria	27	50.58±1.50	93.2%±1.6%	11.72±0.27	92.1%±4.2%
Eutheria	32	50.29±1.29	90.8%±1.9%	11.19±0.23	91.5%±4.5%
Mammals	35	39.36±2.85	76.6%±2.8%	8.64±0.48	73.8%±6.2%

^c^R_i_: Information content/specificity ± 95% confidence interval.

^d^Homology: Average percent ± 95% confidence interval.

## Discussion

The *APP* gene consists of 18 exons and spans more than 300 kilobases on human chromosome 21 [51]. We hypothesize that unusually high production of Aβ may contribute to AD, and this aberrant Aβ production can be a result of unusually high *APP* gene expression. The *APP* gene is a likely locus for sporadic, late-onset AD, the most common form of the disease [2,52,53]. Down syndrome patients invariably develop AD, which may be due to additional gene dose of the *APP* gene in the Down critical region of chromosome 21. In addition, cases of late-onset AD have increased plasma levels of the forms of Aβ [54] specifically associated with the disease. Tissue and cell-type specific expression of the *APP* gene may also play a role in APP protein or Aβ-related pathogenesis. It is well established that mutations in the *APP* coding sequence can cause autosomal dominant early onset FAD [55]. Some analyses of the coding sequence [56] and the promoter [57] rejected other *APP* polymorphisms as predisposing to late onset AD (LOAD). However, there are reports of promoter polymorphisms that increase risk for AD in a non-autosomal fashion [58,59], particularly of two polymorphisms in the APP promoter associated with AD pedigrees [23].

In addition, the following results strongly implicate the role of promoter elements in AD. 1) In a genome screen of 292 sib-pairs with LOAD, Hardy’s group identified 12 loci with lod-scores >1 including a region on chromosomes 21 [60]. 2) Using non-parametric linkage methods, Duke’s group showed that a locus predisposing to LOAD might reside in this region of chromosome 21 [61]. 3) Levels of Aβ correlate to cognitive decline in predementia and dementia [62]. 4) Genetic analysis of a case of DS due to non-disjunction suggested triplication of *APP* in the pathogenesis of AD in Down syndrome [63]. 5) Younkin’s group analyzed plasma Aβ in 180 first-degree relatives of LOAD patients and in 82 age-matched controls. This analysis showed highly significant increase in plasma Aβ40/42 in the relatives as compared to controls [64,65].

Serial deletion analysis of the *APP* promoter has revealed several regulatory elements [14,17]. The *APP* promoter is regulated by several factors, including but not necessarily limited to nerve growth factor, fibroblast growth factor, and interleukin-l [20,21]. Copper depletion downregulates *APP* promoter activity [22]. Various groups have reported other transcriptional elements within the first 100 nt from TSS of the *APP* gene [19,34,66,67]. This includes regulation of *APP* promoter expression by two GC-elements [67], the zinc finger protein CTCF [66], and upstream stimulatory factor [19].

We previously reported discovering a 30 nt (−76/−47) fragment from a deletion series of the *APP* promoter with different effects depending upon cell line [27]. Our discovery was independently paralleled by other workers, who have noted a fragment of the *APP* promoter at −55/−33 by DNAse protection assay. This fragment was predicted to contain AP1 and AP4 binding sites, but both were experimentally excluded [15]. Furthermore, deletion of a −54/−42 fragment of the *APP* promoter resulted in reduced expression of reporter fusion clones [68]. In addition, deletion of a −77/−47 fragment resulted in reduced reporter fusion expression in rat primary embryonic hippocampal neurons [69], which was similar to our own observation of the effect of deleting the PRE in rodent cell lines and opposite what we observed in human cell lines [27]. This other work essentially concluded that USF was the responsible agent for regulation through this *APP* promoter fragment, although it did propose but not test a putative SP1 binding site. We do not dispute that USF may play an important role in *APP* proximal protein regulation but investigate the possibility that other factors and cofactors also operate on this sequence.

We sought to more fully characterize the activity of the PRE as a component of the 5^′^-flanking region of the *APP* gene. Transcription factor binding activity was studied by EMSA with human tissue nuclear extracts, including extracts from human brain, followed by EMSA in four different cell line nuclear extracts under different conditions of stimulation or induction. Binding of specific TFs was tested by competition EMSA and by antibody-supershift EMSA. We also used Southwestern blotting to estimate molecular weights of interaction partners with the PRE and compared these with candidate transcription factors.

We have determined that the PRE has tissue specificity, showing greater DNA-protein interaction with brain and lung extracts than with liver or heart extracts. In addition, the PRE has cell line-specific DNA-protein interactions with cell nuclear extracts, differing among U937, HeLa, U373, PC12, NIH3T3 cell lines. These interactions are subject to reduction, elimination, or qualitative change by conditions such as hypoxia, SR, and induction with TPA or IFN-γ), IL6, or TGFβ. Our results suggest that the −76/−47 region binds to a protein that is upregulated in serum starvation, and downregulated in hypoxia. Because serum starvation contributes to the induction of apoptosis, these results suggest a role of the 30-nt proximal *APP* promoter element in enhanced apoptotic neuronal cell death.

Competition EMSA and antibody-supershift EMSA confirmed predicted interactions between the PRE and AP2, while antibody-supershift EMSA confirmed interaction between the PRE and AP2, PuF, SP1, and USF2. Competition and supershift EMSA appear to contradict each other regarding SP1. Possible biological explanations could include sufficiently high affinity within the PRE for SP1 to overcome competition with the particular commercial probe used. High PRE-SP1 affinity could result in a “negative” competition result but a positive supershift, since the supershift is meant to detect SP1 when bound to the target DNA. Alternatively, our supershift may be interacting with an SP1-like transcription factor that binds the PRE but has a lower affinity with the consensus SP1 site in the commercial competition oligomer. Southwestern blotting indicated possible interactions between the PRE and AP2, SP1, and USF2, among other TF. It also admitted the possibility of PRE-PuF dimer interaction.

We mutated the PRE and highlighted a specific effect of the sequence that included the predicted PuF/SP1 binding site. Our mutant expression evidence tends to agree with our EMSA evidence. Of the mutants characterized, M1 was the most consistent in response across NB and PC12x cells and nuclear extracts. Specifically, semi-quantitative EMSA with M1 probe resulted in significant signal loss at the band position that corresponded closely to the major band of wildtype PRE EMSA in both NB and PC12 nuclear extracts. Likewise, M1 mutant promoter/reporter expression clones resulted in significantly reduced levels of CAT reporter protein in both NB and PC12 cells. The M1 mutant deleted the predicted PuF site and both predicted SP1 sites in the PRE.

We note that M2 and M4 each also deleted a single predicted SP1 site, the specific site differing between M2 and M4. EMSA analysis of these two mutants showed that M4 consistently had reduced EMSA interaction at band II, while M2 did not. However, CAT reporter levels were not consistently altered between cell types. There-fore, our mutation analysis confirms our earlier conclusion that the PRE is most likely to function as a site of PuF and SP1 activity. Disruption of the shared PuF/SP1 site in our system resulted in reduced levels of reporter gene product, which appears to contradict part of our proposed model. However primary sequence disruption of the site would influence both PuF (repressive) and SP1 (stimulatory) interaction very strongly. In addition, disruption of the AP2 site also resulted in loss of EMSA signal, suggesting that the PRE may also function under induction as well as constitutively.

Currently, two DNA sequence polymorphisms have been reported for the PRE, specifically rs200621906 and rs201592736 [37]. Each is predicted to alter potential TF binding sites within the PRE (Table 7). Of particular note, rs201592736 may interfere with the PuF site. In addition, potentially interesting sites were created, including glucocorticoid/progesterone receptor (GR/PR), NF1-like enkephalin nuclear transcription factor 1 (NKTF1) retinoic acid receptor γ (RARγ), and zinc finger E-box-binding homeobox 1 (ZEB1),. Glucocorticoid levels are well associated with AD risk [70]. Progesterone supplementation has been linked to more rapid age-related cognitive decline in post-menopausal women [71,72]. Elevated levels of enkephalin contribute to neurological impairment and tau phosphorylation in AD model animals [73]. Upregulation of enkephalin accompanied by upregulation of APP due to an SNP in the PRE may overwhelm natural defenses against neurodegeneration. RARs direct APP processing toward the non-pathogenic α pathway [74], and it is conceivable that a feedback mechanism that results in RAR-mediated overproduction of APP in a natural PRE mutant may overwhelm this process. ZEB1 interacts with TGFβ [75], and TGFβ is implicated in AD pathogenesis [76,77]. The rs201592736 SNP approximates our M2 mutant, while rs200621906 approximates M4. Combining both polymorphisms approximates the TF deletion effects of M1, which showed significant Bonferroni-adjusted reductions in both EMSA and CAT reporter by meta-analysis. Population frequency of these SNPs has not been published, and it has not been yet determined if they occur as a haplotype or independently. It should be cautioned that, although they have interesting potential TF site changes, functional association with any disease state has also not been determined. 

**Table 7:** Natural SNP in the PRE

**SNP**	**Sequence**	**Sites deleted**	**Sites created**
rs201592736	5’-TCGGGTGCCGAGCGAGGTGGGCCGGATCAG-3’	AP2, GC Box (I)^a^, GATA1, PuF	RARγ, ZEB1
rs200621906	5’-TCGGGTGCCGAGCGGGGTGGGCCAGATCAG-3’	AP2, GC Box (II), SP1 (II)^b^	ENKTF1, GR/PR,
both	5’-TCGGGTGCCGAGCGAGGTGGGCCAGATCAG-3’	AP2, GC Box (both), PuF, SP1 (II)	ENKTF1, GR/PR, RARγ, ZEB1

^a^Two distinct GC boxes were identified in the wildtype PRE sequence and are designated as “I” and “II”.

^b^Two distinct SP1 sites in addition to the GC boxes were identified in the wildtype PRE sequence and are designated as “I” and “II”.

The PRE is strongly conserved in placental mammals, particularly the PuF/SP1 overlapping site. In addition, this conservation occurs in a promoter region that has undergone significant non-neutral (*i. e.*, non-molecular clock) change. In particular, primate and mouse/rat sequences diverge the most from each other in our root-to-tip ana-lysis, but with far less difference when considering the PRE, specifically. We propose that this particular short segment of the *APP* proximal regulatory region has been specifically maintained, as would be expected of an active promoter segment. We have previously stated that other AD-associated gene promoters, such as for *APOE*, have important differences between mouse and human [25]. Likewise, we have reported both differences and similarities between human and rat cell line responses to functional promoter deletion clones of the human *MAPT* promoter [26]. Such overall differences can be put to use in identifying potentially critical regions of similarity, such as the PRE within the *APP* promoter.

Although two SNPs have been reported in the human PRE, it is unlikely, given their obscurity in the literature, that these would be common enough to explain a large proportion of sporadic AD. We propose that the majority of etiologically important PuF vs. SP1 disruption in the PRE would be due to environmental influences on epigenetic markers. The naturally-occurring SNPs could serve as a potential test bed, given their similarity in terms of TF site disruption to some of our synthetic mutants. It is potentially more interesting that the Puf/SP1 site consists primarily of GG and GGG sequences, which are particularly vulnerable to DNA oxidation [78]. While SP1 activity is sensitive to epigenetic DNA modification [79], and PuF operates in repair of DNA damage [80], relative effects of DNA oxidation upon each protein’s activity as a transcription factor have not been established. Likewise, CpG dimers (sites of cytosine methylation) are immediately adjacent to the PuF/SP1 site. Thus, in our system, while disrupting both PuF and SP1 activity by primary sequence mutation may result in overall reduction in reporter levels, environmentally-induced epigenetic alterations to the shared target DNA sequence may favor SP1 stimulation over PuF inhibition. In light of the knowledge that overall PuF activity may be reduced in AD, we do not suggest that epigenetic modification of the PRE would be the sole cause of the disorder. Rather, it would contribute to disruption of normal *APP* gene expression that would accompany AD. Feedback mechanisms between *APP* and other misregulated AD associated genes may serve to also reduce PuF activity in concert with direct disruption of *APP*-PuF interaction. We admit that our current assignment of PuF and SP1 to active status within the PRE has not yet been directly tested.

Mammalian PuF is associated with inhibition of invasive metastasis [81-84] and stimulation of normal cell proliferation [85,86]. PuF kinase activity was reduced in human brain in Alzheimer’s disease and Down syndrome [87]. Total APP mRNA is elevated in AD brain, specifically KPI(+) isoforms [88]. *APP* gene expression and SP1 activity are increased in aging primate brains [31]. SP1-DNA interaction and *APP* expression in mouse brains are increased in a latent fashion by early-life dietary administration of Pb [29]. We suggest an antagonistic role between PuF and SP1, specifically that PuF may serve to downregulate *APP* gene expression via the PRE *in vivo*, and increases in effective SP1 activity, due to increased SP1 levels, interference in PuF-DNA interaction at the PRE, or decrease in PuF levels, for example, may overwhelm PuF regulation.

The PuF transcription factor/kinase was discovered in the context of inhibiting metastasis [28]. Such inhibition included through interaction with the *MYC* gene promoter [89,90]. Later, PuF was determined to also function as a kinase independently of its TF activity [91]. PuF TF activity was determined to not only act to inhibit metastasis, but to also operate in non-metastatic cell proliferation [85,86]. In addition to cell proliferative activity, PuF regulates insulin secretion from pancreas islet cells [92], providing another molecular link underlying the recently-proposed metabolic-cognitive syndrome that may unite metabolic and neurodegenerative disorders [93]. We propose that these aspects of PuF may prove important in understanding both AD pathogenesis and the possibility that a role may be played by *APP* gene products in oncogenesis. Of greater interest is that PuF has already been indirectly linked to AD before our work with the PRE. PuF interacts with calcium/calmodulin-dependent protein kinase II inhibitor 1 (CAMK2N1) [94]. CAMK2N1 is, itself, a potent inhibitor of calcium/calmodulin-dependent protein kinase II (CAMK2), which is a mediator of learning and memory [95]. CAMK2 is currently a target of several anti-AD drugs under investigation [96].

Of particular note is that the PRE exists within a DNAse I hypersensitive region [97], and PuF activity is modulated by the presence or absence of a DNAse I hypersensitive region [87]. Likewise, among its amazing variety of functions [98,99], the APP protein is a ferroxidase and activates ferroportin to export iron from neurons [100]. Iron chelation reduces APP protein levels through the APP mRNA 5’-UTR [101], and this is reversed by iron supplementation. Conversely, overexpression of APP results in reduced iron content and increased oxidative stress in human neuroblastoma cells [102]. Although it has also been shown that abnormally high levels of iron accumulate in specific brain regions in AD, particularly within and around amyloid plaques [103], this is not paradoxical, since plaque-associated iron would be extracellular and could be in part due to overactivity of APP in iron transport. In addition, faulty iron metabolism has been strongly linked to cancer and neoplasia, specifically through ferroportin activity [104], and increased serum iron is associated with greater risk for oxidative damage to DNA [105]. Of particular interest for a possible oncology/*APP* association is that expression of the *APP* gene in response to iron chelators distinctly differed between neoplastic and normal cell lines [106]. In cancer patients, increased *APP* gene expression was more common in tumor tissue of oral squamous cell carcinoma patients than in non-cancerous matched tissue samples. More importantly, in those patients who had elevated *APP* expression, survival at 24 months was 48% vs. 82% for patients with non-elevated *APP* expression [107]. This suggests that PuF may exert anti-metastatic properties by, in part, altering levels of *APP* expression, reducing overall results of APP protein’s ferroxidase activity and modulating cancer risk.

Basal activity of the *APP* promoter is greatest in neural origin cell lines, and several promoter elements close to the PRE are important for this activity [108]. For example, the *APP* promoter contains binding sites for important TFs, such as purine-rich element binding protein-α (PURα) [109] and early growth response 1 (EGR1) [110]. Notably, there is a high expression of PURα and EGR1 in neurons during brain development and in the adult brain, and negative regulation of APP gene expression by PURα [109].

## Conclusions

Interference with TF binding to the PRE in the context of the complete *APP* promoter could serve to deregulate *APP* production. Deletion of the PRE from a complete series of *APP* promoter clones resulted in a 3 to 4 fold gain of promoter activity in NB cells as measured by levels of reporter gene product [27]. While AP2 and USF2 (implicated in our antibody supershifts) are generally stimulatory factors, the normal function of PuF is usually inhibitory of pathogenic cell proliferation, specifically metastasis [83,84].

**Figure 10:**
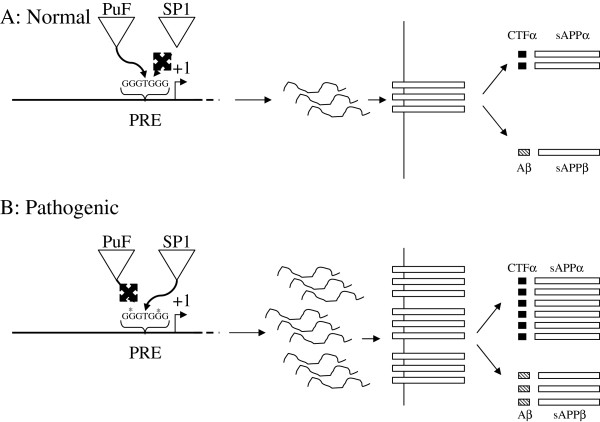
**Model of Antagonistic PuF vs. SP1 regulation of the *APP *gene and disruption in AD. A**. Non-pathogenic APP expression, levels regulated by PuF. In normal brain, PuF would compete with SP1 at the PRE and result in normal levels of APP mRNA and protein. Aβ would be restricted to non-pathogenic concentrations. **B**. Pathogenic APP expression resulting from disruption of PuF regulation of the PRE. Disruption of the PRE, such as through environmentally induced DNA oxidation at GG dimers, “*”, may reduce affinity of the PRE for PuF more severely than for SP1. Effective SP1 activity then serves to upregulate APP expression levels beyond a pathogenic threshold.

In addition to inhibiting metastasis, PuF serves to induce normal cell proliferation [83,84]. The APP protein, among its non-pathological functions, is active in the “neuronal pruning” stage of brain development, wherein neurons in the early brain are selectively removed after rapid proliferation [111]. In addition to neurogenesis, PuF homologues regulate early morphological development [112]. Thus, as PuF serves to stimulate non-pathogenic cell proliferation, and APP can act to reduce cell proliferation, it is reasonable to propose the possibility that PuF acts to downregulate *APP* expression under normal circumstances, and interference in PuF regulation through the PRE could serve to deregulate *APP* gene expression, potentially contributing to AD pathogenesis (Figure 10). Aβ is a minority product of *APP* processing [113]. Therefore, increase in *APP* levels could raise Aβ above a risk threshold for AD. A theoretical union of oncogenic and neurodegenerative models has been proposed in the latent early-life associated regulation (LEARn) model of idiopathic neuropsychiatric disorders [38,39], expands and extends the “*n*-hit” model of pathogenesis commonly accepted in oncology to “sporadic” neurodegenerative disorders. Given that the predicted PuF site may be structurally vulnerable to GG oxidation, and thus subject to the LEARn model, we conclude that our work suggests connections between processes that lead to cancer etiology and sporadic neurobiological disorders, both conceptually (LEARn vs. *n*-hit) and mechanistically (PuF regulation of *APP* vs. PuF regulation of metastasis). 

## Methods

### Reagents and enzymes

Unless specifically noted otherwise, reagents were purchased from Sigma-Aldrich (St. Louis, MO), and enzymes were purchased from New England Biolabs (Ipswich, MA) or Roche Life Science (Indianapolis, IN).

### Cell culture

PC12 and NB cell lines were obtained from ATCC and cultured as described previously [114]. The plates were incubated in 5% CO_2_ in a 37°C incubator. Cell culture reagents were purchased from Invitrogen/Life Technologies (Carlsbad, CA).

### PRE fragments and oligomers

**Figure 11:**
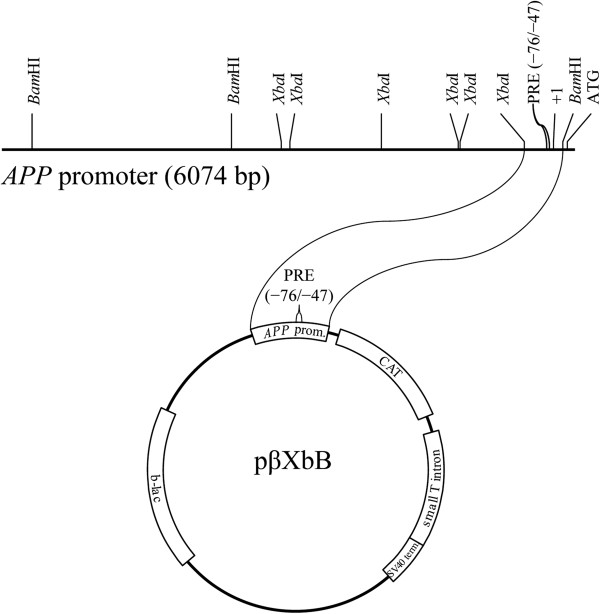
**Clone pβXbB.** A deletion series of 6kb of the APP promoter and 5′-UTR was previously constructed [17]. Within this series, an XbaI/BamHI 408bp fragment of the APP proximal promoter and 5′-UTR was inserted in the expression vector pBLCAT3 (Promega). This fragment contained the PRE. The clone was used as the base template for all mutagenesis described herein.

The rhesus monkey *APP* promoter clone pβXbB [17] (Figure 11) was digested with restriction enzymes PvuII and XhoI and run on a 10% TAE-polyacrylamide gel. The cell type specific 30 nt (−76/−47) PRE [27] was purified and end-labeled with γ^32^P-ATP (Amersham, Piscataway, NJ) by T4 polynucleotide kinase (Roche, Indianapolis, IN). In addition, oligomers corresponding to this sequence and its reverse complement were synthesized (Invitrogen) and similarly radiolabeled. The oligomer pair differs from the plasmid fragment in that it does not have a “TCGA” 5^′^ overhang. This overhang was filled for wildtype and mutant (see “*Semiquantitative EMSA of mutant PRE oligomers*…”, herein) synthetic double-stranded oligomers. 

### Analysis of potential transcription factor binding sites on the PRE

The TESS [40] and MatInspector [41] utilities were used to probe the TransFac database with the PRE sequence.

### EMSA of PRE fragments in human tissue nuclear extracts

Cell nuclei extracts from human brain, heart, liver, and lungs were prepared as previously described [115,116]. The assay was carried out with 40 pg of PRE from digestion of pβXbB (about 10,000 cpm) and 10 to 20 μg of nuclear extracts. Radioactive probe was incubated with human brain, heart, liver, or lung nuclear extracts in 24μl of EMSA-binding buffer (Active Motif, Carlsbad CA) at 6°–8°C for 40 minutes. The samples were mixed with loading dye and the products of the reaction were separated on a 5% nondenaturing polyacrylamide gel electrophoresis (PAGE) in 1 x TGE (50 mM Tris, 384 mM glycine, and 2 mM EDTA). The gel was dried and exposed to X-ray film for autoradiography. Free oligonucleotides ran at the bottom of the gel. Protein-DNA complexes were detected as mobility-retarded bands.

### EMSA in nuclear extracts from normal and variously stimulated PC12 and human U937 cells

Nuclear extracts were obtained commercially (ActiveMotif). PRE oligomer pairs were annealed and radiolabeled as described herein. Radioactive oligomer pairs were incubated with nuclear extracts from PC12, hypoxic PC12, U937, and U937 stimulated with either IFN-γ, TPA, or IFN-γ + TPA. In addition, one sample was incubated with U937 nuclear extracts and 200x molar excess of unlabeled PRE oligomer pairs. Reaction mixtures were analyzed on nondenaturing PAGE and the gel used for autoradiography.

### Competition EMSA in nuclear extracts from NB and HeLa cells

Cell nuclear extracts from NB and either HeLa cultures or HeLa treated with TPA were obtained commercially (Active Motif). Oligomer pairs for the PRE were annealed and radiolabeled as described herein. Nuclear extracts and oligomer pairs were incubated as described herein, except that some individual samples were incubated with 200x molar excess of unlabeled oligomer pairs. NB extracts were either uncompeted or competed against unlabeled PRE or commercially-obtained oligomer pairs (Santa Cruz Biotechnology, Santa Cruz, CA) known to bind AP1, AP2, SP1, or USF1. EMSA in unstimulated HeLa extracts was not subject to competition. Extracts from TPA-stimulated HeLa were either uncompeted or competed against unlabeled PRE or commercially-obtained oligomers known to bind AP1, AP2, or USF1. Reaction products were separated on 5% nondenaturing PAGE and used for autoradiography as described herein.

### Antibody-supershift EMSA of the PRE in NB extracts vs. several antisera

PRE oligomer pairs were annealed and radiolabeled as described herein. Labeled oligomers were incubated with NB nuclear extracts. Reactions were carried out either without antiserum or separately with antiserum against the nuclear factors AP1, AP2, SP1, PuF, USF1, or USF2 (Santa Cruz Biotechnology). In addition, one reaction was incubated with both of the two antisera against AP2 and PuF. Reactions were analyzed on nondenaturing 5% nondenaturing PAGE and the gel subject to autoradiography.

### Southwestern hybridization of the PRE vs. nuclear extracts from NB, HeLa, TPA-stimulated HeLa, and PC12 cells

Nuclear extracts from NB, HeLa, TPA-stimulated HeLa, and PC12 cells (5μg of each) were subject to denaturing PAGE on a 10% gel containing 0.1% SDS. Proteins were transferred to 0.2μm nitrocellulose membranes and probed with radiolabeled PRE as we have previously described [117]. Briefly, membranes were incubated overnight at 4°C in renaturation buffer (10 mM HEPES pH 7.9, 50 mM NaCl, 0.1 mM EDTA 10 mM MgCl_2_, 10% glycerol (v/v), 5% dry skim milk (w/v). Hybridization was done in the same buffer reducing skim milk to 0.25% and adding 10 μg poly-dI:dC and 10^6^ CPM/ml radiolabeled probe. Membranes were incubated with probe for 24 hours and washed twice in 50 ml of hybridization buffer lacking poly-dI:dC and probe, 15 minutes per wash, followed by autoradiography.

### Mutagenic studies of the PRE sequence, design of PRE mutants

**Figure 12:**
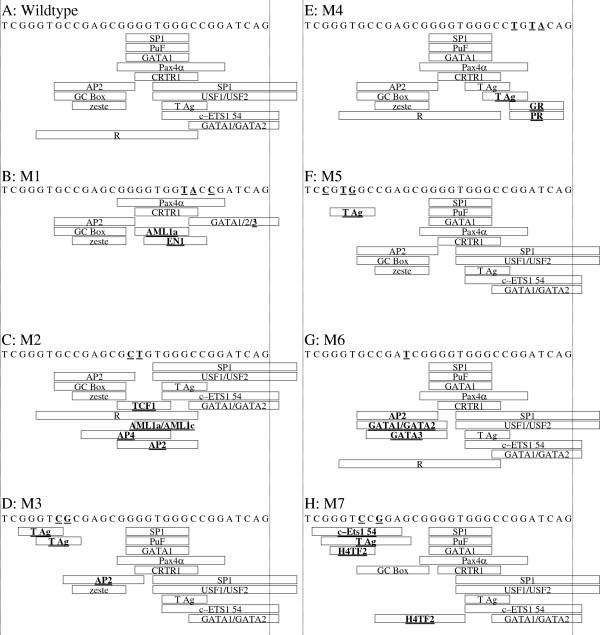
**PRE mutant designs.** TESS predictions of transcription factor binding sites (Table 1) were used to design oligomers for deleting one or more putative transcription factor binding sites in the PRE. ***A.*** Wildtype PRE sequence with all target sites indicated by rectangles corresponding to TF positions. ***B-H.*** Mutant oligomers with specific target sites indicated by rectangles corresponding to TF positions. TF sites introduced by mutagenesis have boldface and underlined names. Specific base mutations indicated by boldface and underline in sequences.

The PRE sequence was used as a template to design mutants for selected predicted transcription factor binding sites (Figure 12). Two sets of complementary oligomer pairs were synthesized (Invitrogen) according to these designs. The first set consisted of oligomers that were mutated versions of the PRE for EMSA studies (Table 8). The second set consisted of oligomers for generating site-directed mutant CAT fusion clones (Table 9), based on the pβXbB [17]*APP*-CAT fusion clone. This parent vector contains a 408nt proximal fragment of the *APP* gene promoter and 5^′^-UTR (Figure 12). The TransFac database was probed with TESS [40] and MatInspector [41] to determine specific changes in potential TF binding sites. Each mutant deleted specific predicted TF binding sites and incidentally created other TF sites (Table 10). 

**Table 8:** Oligomers for EMSA of PRE-derived mutants

**Oligomer**	**Forward (F) 5′ to 3′**^**a**^	**Reverse (R) 5′ to 3′**
PRE (WT)	5^′^-TCGAGTGCCGAGCGGGGTGGGCCGGATCAG-3^′^	5^′^-CTGATCCGGCCCACCCCGCTCGGCACTCGA-3^′^
M1	5^′^-TCGAGTGCCGAGCGGGGTGGTACCGATCAG-3^′^	5^′^-CTGATCGGTACCACCCCGCTCGGCACTCGA-3^′^
M4	5^′^-TCGAGTGCCGAGCGGGGTGGGCCTGTACAG-3^′^	5^′^-CTGTACAGGCCCACCCCGCTCGGCACTCGA-3^′^
M2	5^′^-TCGAGTGCCGAGCGCTGTGGGCCGGATCAG-3^′^	5^′^-CTGATCCGGCCCACAGCGCTCGGCACTCGA-3^′^
M3	5^′^-TCGAGTCGCGAGCGGGGTGGGCCGGATCAG-3^′^	5^′^-CTGATCCGGCCCACCCCGCTCGCGACTCGA-3^′^
M4	5^′^-TCCATGGCCGAGCGGGGTGGGCCGGATCAG-3^′^	5^′^-CTGATCCGGCCCACCCCGCTCGGCCATGGA-3^′^
M6	5^′^-TCGAGTGCCGATCGGGGTGGGCCGGATCAG-3^′^	5^′^-CTGATCCGGCCCACCCCGATCGGCACTCGA-3^′^
M7	5^′^-TCGAGTCCGGAGCGGGGTGGGCCGGATCAG-3^′^	5^′^-CTGATCCGGCCCACCCCGCTCCGGACTCGA-3^′^

^a^Underlined bases have been mutated from wildtype PRE.

**Table 9:** Oligomers for site-directed mutagenesis of the pβXbB PRE/CAT fusion plasmid

**Oligomer**	**Forward (F) 5′ to 3′**	**Reverse (R) 5′ to 3′**	**RE**^**a**^
M1m-F	5^′^-CCGAGCGGGGTGGTACCGATCAGCTGACTTGCC-3^′^	5^′^-GGCAAGTCAGCTGATCGGTACCACCCCGCTCGG-3^′^	*Kpn*I
M4m-F	5^′^-CGAGCGGGGTGGGCCTGTACAGCTGACTTGCC-3^′^	5^′^-GGCAAGTCAGCTGTACAGGCCCACCCCGCTCG-3^′^	*Bsr*GI
M2m-F	5^′^-TCGAGTGCCGAGCGCTGTGGGCCGGATCAG-3^′^	5^′^-CTGATCCGGCCCACAGCGCTCGGCACTCGA-3^′^	*Afe*I
M3m-F	5^′^-CTGGATCTCGAGTCGCGAGCGGGGTGGGC-3^′^	5^′^-GCCCACCCCGCTCGCGACTCGAGATCCAG-3^′^	*Nru*I
M4m-F	5^′^-CCAGATCTGGATCTCCATGGCCGAGCGGGGTGGG-3^′^	5^′^-CCCACCCCGCTCGGCCATGGAGATCCAGATCTGG-3^′^	*Nco*I
M6m-F	5^′^-CTCGAGTGCCGATCGGGGTGGGCCG-3^′^	5^′^-CGGCCCACCCCGATCGGCACTCGAG-3^′^	*Pvu*I
M7m-F	5^′^-CTGGATCTCGAGTCCGGAGCGGGGTGGGCCG-3^′^	5^′^-CGGCCCACCCCGCTCCGGACTCGAGATCCAG-3^′^	*Bsp*EI

^a^RE: Restriction enzyme introduced.

^b^Mutated oligonucleotides are underlined.

**Table 10:** Mutations induced in the PRE

**Variant**	**Sites deleted**	**Sites created**
Wildtype PRE		
M1	AP2, GATA1, GC Box (II)^a^, PuF, R, SP1 (I)^b^	AML1a
M2	AP2, GC Box (I), GATA1, Pax4α, PuF, SP1 (I)	AML1a/AML1c, AP4, GT-IIBα
M3	R, TCERG (I)^c^	
M4	GATA1/GATA2, SP1 (II)	GR, PR
M5	R, TCERG (I)	YY1
M6	TCERG1 (II)	GATA1/GATA2
M7	TCERG1 (I, II), R	

^a^Two distinct GC boxes were identified in the wildtype PRE sequence and are designated as “I” and “II”.

^b^Two distinct SP1 sites in addition to the GC boxes were identified in the wildtype PRE sequence and are designated as “I” and “II”.

^c^Two distinct TCERG1 sites were identified in the wildtype PRE sequence and are designated as “I” and “II”.

### Semiquantitative EMSA of mutant PRE oligomers with NB and PC12 extracts

Oligomer pairs of wildtype PRE and 7 mutants (Table 8) were commercially obtained (Invitrogen), annealed, and radiolabeled. The labeled double-stranded oligomers were used in EMSA reactions with NB and PC12 nuclear extracts, as described herein. Gels were used for autoradiography. The autoradiographs were densitometrically scanned and the scans quantified with ImageJ analysis software [118]. Both band density and position were measured. Dunnett’s two-tailed *t* test was used to analyze both density and migration differences of each mutant compared to wildtype PRE.

### Mutagenesis of the PRE sequence in the pβXbB CAT reporter clone

Mutagenic oligomers (Table 9) were designed and synthesized (Invitrogen). These oligomers were used with the QuikChange site-directed mutagenesis kit (Stratagene, CA) to insert specific mutations into the PRE sequence within the *APP* promoter/CAT reporter fusion clone pβXbB (Figure 12). Mutants were initially screened by digestion with designed restriction enzyme sites (Table 9) and confirmed via DNA sequencing (Macrogen Korea, Seoul, South Korea).

### Transfection of NB and PC12 cells

Cells were transfected with wildtype and mutated CAT reporter constructs by the Lipofectamine plus kit (Invitrogen). Briefly, transfection was carried out with 2.7 μg of DNA of each CAT-reporter plasmid. To monitor transfection efficiency, cells were cotransfected with 0.3 μg pSVβGAL (Promega). Following DNA transfections, cells were harvested, cell extracts prepared, and protein concentration determined by Bradford assay (BioRad, Hercules, CA).

### Functional assay of pβXbB and mutant CAT reporter clones by ELISA

Reporter CAT protein levels in cells transfected by wildtype and each mutated clone were measured with an ELISA kit (Roche, Indianapolis), and CAT protein levels were adjusted to total protein content of extracts. Each assay was performed as described previously [114]. Results from adjusted reporter gene activity were statistically analyzed via Dunnett’s multiple *t*.

### EMSA of selected PRE mutants in nuclear extracts from different cell lines and from cell lines subjected to hypoxia, serum starvation, or induction with different cytokines

Nuclear extracts from HeLa; HeLa cultured with TPA; NB; NIH3T3; NIH3T3 cultured under serum starvation (SR), IL6, or TGFβ; PC12; PC12 cultured under hypoxia; U373 cell lines; human brain; or mouse brain were obtained commercially (ActiveMotif) or prepared as previously described [119]. Oligomer pairs for the PRE and for mutants M1, M4, and M6 (Table 8) were annealed and radiolabeled and oligomers and extracts were incubated, electrophoresed on 5% native TGE-PAGE, and gels used for autoradiography as described herein.

### Statistical analysis

All statistical analysis was done with the R statistical language [120]. Data were checked by Anderson-Darling test for normality of residuals and non-linearly transformed if necessary. Each mutant’s mean adjusted signal was compared to wildtype by Dunnett’s multiple *t*[121], p ≤ 0.05. Root mean square standardized effect [122] with sample size bias adjustment was calculated for each assay with the equation , and unbiased standardized pairwise mean differences (*g*) between wildtype PRE and each mutant within each assay were also calculated with the equation $g=\frac{m_{j}-m_{c}}{\sqrt{\mathrm{MSerror}}}\times \frac{\Gamma \left(\frac{2n-2}{2}\right)}{\Gamma \left(\frac{2n-3}{2}\right)\sqrt{\frac{2n-2}{2}}}$[43]. Symbols in these equations are explained in Table 11. The Ψ standardized effect size has a comparable range to *g*, since Ψ expresses effect size in $\sqrt{\mathrm{MSerror}}$ of the ANOVA, which is analogous to standard deviation of a single pairwise comparison. 

**Table 11:** Symbols in effect size equations

**Symbol**	**Use**
*g*	unbiased standard difference
GM	grand mean of experiment
*k*	total groups of test
m_c_	mean value of PRE wildtype control
m_j_	mean value of group j
MSerror	mean square error of ANOVA
*n*	replicates within each group
Γ()	gamma function
Ψ	root mean square standard effect

### Interspecies analysis of the PRE

Genomic sequences identified or preliminarily identified as proximal promoter elements of the *APP* genes of 35 mammal species (Table 12) were downloaded from NCBI/GenBank. Sequences were aligned by *WebPrank*[44], and the alignment was trimmed at each end. The resulting alignment covered −326/+202 in the human sequence. Taxonomically weighted mean information content was calculated for a 100 nt window, along with 95% confidence intervals, in the alignment, ignoring any region that was a gap within the primates. In addition, the portion of the alignment corresponding to the PRE sequence was used to calculate taxonomically weighted homologies and sequence logos [45]. “Base weights” were assigned on the basis of each clade equally as it joined to the taxonomic guide tree as a whole. These “base weights” were then converted by the equation $2^{\frac{1}{\mathrm{baseweight}}}$ into “working weights”. Information content was then multiplied by individual working weights before combination with other taxons. The working weights were also adjusted within each analysis so that the highest-value working weight was equal to 1. This process was meant to partially compensate for “over-representation” of some phylogenic groups vs. others in the analyses. 

**Table 12:** Sequences for interspecies PRE sequence comparison

**Species**	**Abbreviation**	**Common name**	**Sequence**	**Loc.**^**a**^	**Base weight**^**b**^
*Homo sapiens*	*Hom sap*	human	[GenBank:NT_011512.1]	13204753.13205282	11
*Pan troglodytes*	*Pan tro*	chimpanzee	[GenBank:NT_106996.1]	12354873.12355401	11
*Gorilla gorilla*	*Gor gor*	western gorilla	Ensembl:gorGor3.1:21	14453005.14453533	10
*Pongo pygmaeus*	*Pon pyg*	Bornean orangutan	[GenBank:NW_002891078.1]	100309.100838	10
*Nomascus leucogenys*	*Nom leu*	northern white-cheeked gibbon	[Genbank:NW_003501402]	11777748.11778275	7
*Macaca mulatta*	*Mac mul*	rhesus monkey	[GenBank:NW_001114168.1]	7908454.7908980	7
*Callithrix jacchus*	*Cal jac*	common marmoset	[GenBank:NW_003184659]	4142710.4143249	6
*Tarsius syrichta*	*Tar syr*	Philippine tarsier	[GenBank:ABRT010367312]	35.605	5
*Otolemur garnettii*	*Oto gar*	greater galago	Ensembl:scaffold:OtoGar3:GL873528.1	10760940.10765774	4
*Cavia porcellus*	*Cav por*	guinea pig	[GenBank:NT_176367]	7334345.7334880	10
*Octodon degus*	*Oct deg*	common degu	[GenBank:AJSA01048215]	19979.20497	10
*Heterocephalus glaber*	*Het gla*	naked mole rat	[GenBank:AFSB01163805]	427.1000	6
*Ictidomys tridecemlineatus*	*Ict tri*	thirteen-lined ground squirrel	[GenBank:AAQQ01741479]	107.624	9
*Mus musculus*	*Mus mus*	house mouse	[GenBank:NT_039625.8]	19878914.19879574	9
*Rattus norvegicus*	*Rat nor*	Norway rat	[GenBank:NW_047354]	24693771.24694300	8
*Dipodomys ordii*	*Dip ord*	Ord’s kangaroo rat	[GenBank:ABRO01075802]	1.581	6
*Oryctolagus cuniculus*	*Ory cun*	European rabbit	[GenBank:NW_003159292]	59870509.59871025	5
*Ochotona princeps*	*Och pri*	American pika	[GenBank:AAYZ01049311]	693.1006	5
*Bos primigenius taurus*	*Bos pri t*	cow	[GenBank:NW_003103795.1]	2852543.2853063	12
*Bos primigenius indicus*	*Bos pri i*	zebu	[GenBank:AGFL01000631]	71936.72456	12
*Tursiops truncatus*	*Tur tru*	bottlenose dolphin	[GenBank:ABRN02546477]	189.717	7
*Sus scrofa*	*Sus scr*	pig	[GenBank:NW_003611878]	681338.681875	6
*Ailuropoda melanoleuca*	*Ail mel*	giant panda	[GenBank:NW_003217675]	561990.562540	8
*Mustela puturius furo*	*Mus put f*	domestic ferret	[GenBank:AEYP01042946]	556.1076	8
*Canis lupus familiaris*	*Can lup f*	dog	[GenBank:NW_003726114]	21614163.21614717	7
*Pteropus vampyrus*	*Pte vam*	flying fox	[GenBank:ABRP01159960]	503.1000	4
*Condylura cristata*	*Con cry*	star-nosed mole	[GenBank:AJFV01010076]	10886.11380	3
*Loxodonta africanus*	*Lox afr*	African elephant	[GenBank:NW_003573433.1]	30067875.30068350	4
*Procavia capensis*	*Pro cap*	rock hyrax	[GenBank:ABRQ01094447]	1072.1437	4
*Echinops telfairi*	*Ech tel*	lesser hedgehog tenrec	[GenBank:AAIY01071949]	2252.2666	3
*Choloepus hoffmanni*	*Cho hof*	Hoffman’s two-toed sloth	[GenBank:ABVD01435293]	1.364	3
*Dasypus novemcinctus*	*Das nov*	nine-banded armadillo	[GenBank:AAGV03159065]	1.297	3
*Macropus eugenii*	*Mac eug*	Tammar wallaby	[GenBank:ABQO020034033]	684.1293	2
*Sarcophilus harrisii*	*Sar har*	Tasmanian devil	[GenBank:AEFK01137980]	744.1216	2
*Mondelphis domestica*	*Mon dom*	gray short-tailed opossum	[GenBank:NW_001581956.1]	6492289.6491655	1

^a^Location within accession numbered database sequence.

^b^Base weight was used to determine specific weights for each analysis as described in the main text.

In addition, maximum likelihood distances for the complete edge-trimmed alignment were generated by TreePuzzle [123]. Gaps were encoded as suggested by Felsenstein [124] and DNAPARS was used to create an input tree, which was used with the distance matrix to estimate phylogeny with FITCH. The tree was artificially rooted between marsupials and eutheria. Root-to-tip distances were calculated for each species and compared to mean root-to-tip distance ± 95% confidence interval, Bonferroni-adjusted for 35 comparisons.

## Abbreviations

AD: Alzheimer’s disease; AP1: Activator protein 1; AP2: Activator protein 2; *APOE*: Apolipoprotein E gene; APP: Amyloid β-precursor protein; *APP*: Amyloid β-precursor protein gene; Aβ: Amyloid β peptide; CAMK2: Calcium/calmodulin-dependent protein kinase II; CAMK2N1: Calcium/calmodulin-dependent protein kinase II inhibitor 1; CAT: Chloramphenicol acetyltransferase; c-ets1 54: Phosphorylated erythroblastosis virus E26 oncogene homolog 1; E2F: E2F transcription factor; EGR1: Early growth response 1; EMSA: Electrophoretic mobility shift assay/gel shift; FBS: Fetal bovine serum; GATA1: GATA binding protein 1; GATA2: GATA binding protein 2; HeLa: Human cervical epithelial cell line; IFN: Interferon; IL6: Interleukin-6; MAPT: Microtubule-associated protein τ gene; NB: Human SK-N-SH neuroblastoma cells/SK-N-BE neuroblastoma extracts; NIH3T3: Mouse embryo fibroblast cell line; PAGE: Polyacrylamide gel; Pax4a: Paired box gene 4-a; PC12: rat pheochromocytoma cells and extracts; PRE: APP gene proximal regulatory element; PuF: nm23 nucleoside diphosphate kinase/metastatic inhibitory protein; PURα: Purine-rich element binding protein-α; R: Epstein-Barr virus transcription factor R; SNP: single-nucleotide polymorphism; SP1: Specificity protein 1; TCERG1: Transcription elongation regulator 1 (mammal homologue to zeste); TGFβ: transforming growth factor β; TPA: 12-O-tetradecanoylphorbol-13-acetate; TSS: Transcription start site; U373: Human glioblastoma-astrocytoma; U937: Human histiocytic lymphoma cell line; USF1: Upstream stimulatory factor 1; USF2: Upstream stimulatory factor 2.

## Competing interests

The authors have no competing interests regarding this paper.

## Authors’ contributions

DKL participated in design of the study, drafting of the manuscript, and had final approval of the version to be published. BM participated in design of the study and oligomers for EMSA, designed and build mutant expression clones, carried out bioinformatic, EMSA, and functional data analysis, and in drafting of the manuscript. JR participated in reading the manuscript critically and provided useful comments. YG participated in design of the study, carried out EMSA and transfection studies, and assays of reporter protein levels. All authors read and approved the final manuscript.
